# Comprehensive profile of the companion animal gut microbiome integrating reference-based and reference-free methods

**DOI:** 10.1093/ismejo/wrae201

**Published:** 2024-10-12

**Authors:** Tobyn Branck, Zhiji Hu, William A Nickols, Aaron M Walsh, Amrisha Bhosle, Meghan I Short, Jacob T Nearing, Francesco Asnicar, Lauren J McIver, Sagun Maharjan, Ali Rahnavard, Artemis S Louyakis, Dayakar V Badri, Christoph Brockel, Kelsey N Thompson, Curtis Huttenhower

**Affiliations:** Department of Biostatistics, Harvard T.H. Chan School of Public Health, Boston, MA, United States; Harvard Chan Microbiome in Public Health Center, Harvard T. H. Chan School of Public Health, Boston, MA, United States; Science and Technology Center, Hill’s Pet Nutrition, Inc., Topeka, KS, United States; Department of Biostatistics, Harvard T.H. Chan School of Public Health, Boston, MA, United States; Harvard Chan Microbiome in Public Health Center, Harvard T. H. Chan School of Public Health, Boston, MA, United States; Department of Biostatistics, Harvard T.H. Chan School of Public Health, Boston, MA, United States; Harvard Chan Microbiome in Public Health Center, Harvard T. H. Chan School of Public Health, Boston, MA, United States; Infectious Disease and Microbiome Program, Broad Institute of MIT and Harvard, Cambridge, MA, United States; Department of Biostatistics, Harvard T.H. Chan School of Public Health, Boston, MA, United States; Harvard Chan Microbiome in Public Health Center, Harvard T. H. Chan School of Public Health, Boston, MA, United States; Infectious Disease and Microbiome Program, Broad Institute of MIT and Harvard, Cambridge, MA, United States; Department of Biostatistics, Harvard T.H. Chan School of Public Health, Boston, MA, United States; Harvard Chan Microbiome in Public Health Center, Harvard T. H. Chan School of Public Health, Boston, MA, United States; Infectious Disease and Microbiome Program, Broad Institute of MIT and Harvard, Cambridge, MA, United States; Department of Biostatistics, Harvard T.H. Chan School of Public Health, Boston, MA, United States; Harvard Chan Microbiome in Public Health Center, Harvard T. H. Chan School of Public Health, Boston, MA, United States; Infectious Disease and Microbiome Program, Broad Institute of MIT and Harvard, Cambridge, MA, United States; Department of Biostatistics, Harvard T.H. Chan School of Public Health, Boston, MA, United States; Harvard Chan Microbiome in Public Health Center, Harvard T. H. Chan School of Public Health, Boston, MA, United States; Infectious Disease and Microbiome Program, Broad Institute of MIT and Harvard, Cambridge, MA, United States; CIBIO Department, University of Trento, Trento, Italy; Department of Biostatistics, Harvard T.H. Chan School of Public Health, Boston, MA, United States; Harvard Chan Microbiome in Public Health Center, Harvard T. H. Chan School of Public Health, Boston, MA, United States; Department of Biostatistics, Harvard T.H. Chan School of Public Health, Boston, MA, United States; Harvard Chan Microbiome in Public Health Center, Harvard T. H. Chan School of Public Health, Boston, MA, United States; Infectious Disease and Microbiome Program, Broad Institute of MIT and Harvard, Cambridge, MA, United States; Computational Biology Institute, Department of Biostatistics and Bioinformatics, Milken Institute School of Public Health, The George Washington University, Washington, DC, United States; Science and Technology Center, Hill’s Pet Nutrition, Inc., Topeka, KS, United States; Science and Technology Center, Hill’s Pet Nutrition, Inc., Topeka, KS, United States; Science and Technology Center, Hill’s Pet Nutrition, Inc., Topeka, KS, United States; Department of Biostatistics, Harvard T.H. Chan School of Public Health, Boston, MA, United States; Harvard Chan Microbiome in Public Health Center, Harvard T. H. Chan School of Public Health, Boston, MA, United States; Infectious Disease and Microbiome Program, Broad Institute of MIT and Harvard, Cambridge, MA, United States; Department of Biostatistics, Harvard T.H. Chan School of Public Health, Boston, MA, United States; Harvard Chan Microbiome in Public Health Center, Harvard T. H. Chan School of Public Health, Boston, MA, United States; Infectious Disease and Microbiome Program, Broad Institute of MIT and Harvard, Cambridge, MA, United States; Department of Immunology and Infectious Diseases, Harvard T. H. Chan School of Public Health, Boston, MA, United States

**Keywords:** companion animals, gut microbiome, metagenomics, metagenomic assembly, host adaptation, microbial evolution

## Abstract

The gut microbiome of companion animals is relatively underexplored, despite its relevance to animal health, pet owner health, and basic microbial community biology. Here, we provide the most comprehensive analysis of the canine and feline gut microbiomes to date, incorporating 2639 stool shotgun metagenomes (2272 dog and 367 cat) spanning 14 publicly available datasets (*n* = 730) and 8 new study populations (*n* = 1909). These are compared with 238 and 112 baseline human gut metagenomes from the Human Microbiome Project 1-II and a traditionally living Malagasy cohort, respectively, processed in a manner identical to the animal metagenomes. All microbiomes were characterized using reference-based taxonomic and functional profiling, as well as de novo assembly yielding metagenomic assembled genomes clustered into species-level genome bins. Companion animals shared 184 species-level genome bins not found in humans, whereas 198 were found in all three hosts. We applied novel methodology to distinguish strains of these shared organisms either transferred or unique to host species, with phylogenetic patterns suggesting host-specific adaptation of microbial lineages. This corresponded with functional divergence of these lineages by host (e.g. differences in metabolic and antibiotic resistance genes) likely important to companion animal health. This study provides the largest resource to date of companion animal gut metagenomes and greatly contributes to our understanding of the “One Health” concept of a shared microbial environment among humans and companion animals, affecting infectious diseases, immune response, and specific genetic elements.

## Introduction

Characterization of the human gut microbiome has improved understanding of a wide range of acute and chronic health conditions [1, 2], and there is a need to do the same for other host organisms. This is particularly true for companion animals (domesticated cats and dogs), for whom health maintenance is similarly important and for which improved microbial diagnostic biomarkers, dietary guidelines, and disease treatments would be especially beneficial. Lifelong monotonous diets mean that nutrient absorption [3–5], diet-linked phenotypes such as obesity [6], and gastrointestinal conditions such as chronic enteropathy [7, 8] are all relevant. However, companion animal microbiomes remain comparatively underexplored, with larger proportions of uncharacterized microbes [3, 9], making it challenging to manage the health and chronic conditions of companion animals [10, 11] and to determine how they relate to those of pet owners.

Previous studies of canine and feline microbiomes have been largely motivated by applications such as the response to commercial pet foods, with special attention to dietary modifications favoring microbial community compositions that mitigate inflammatory enteropathies [12], obesity [5, 13, 14], and renal disease [15, 16]. For instance, chronic kidney disease (CKD) is one of the most common pathologies in companion animals, and recent work has shown that diets with different macronutrient sources for its management elicit distinct changes to the gut microbiome in both healthy and CKD animals [16, 17]. The goals of such research are generally distinct from those of microbiome studies in agricultural livestock, which instead optimize targets such as production [18], methane emissions [19, 20], or zoonoses [21, 22]. Both application areas, however, emphasize the degree to which variation in the animal gut microbiome correlates with animal health.

Both of these application areas also influence human microbial health. Humans are closely associated with companion animals and indirectly associated with other domesticated animals, such as livestock, by proximity and through the consumption of animal and (fertilized) plant products; both types of exposure foster transmission of enteric microbes. A significant body of research has focused on zoonotic diseases carried by domesticated animals. Enteric pathogens, such as *Clostridioides difficile*, *Campylobacter* spp., and virulent *Escherichia coli*, can all be transmitted from pets to pet owners [23–25]. Additionally, horizontal gene transfer occurs between captive or wild animal and human gut microbiomes, and this includes the transfer of factors such as antimicrobial resistance [26–28]. In some cases, this can specifically affect pathogens such as methicillin-resistant *Staphylococcus aureus* as transmitted from livestock to humans [29]. Similarly, zooanthroponotic transmission of pathogenic strains can conversely occur from humans to domesticated and wild animals [30–32]. All of these examples emphasize the degree to which the microbial health of humans is interrelated to that of other mammalian hosts.

Relatively little is known about the health implications of commensal transmission between pets and humans (i.e. transmission of non-overtly pathogenic microbes). Prior studies reported that differences in both diversity of the human gut microbiome and the abundance of certain taxa depend on pet exposure [33, 34]. There is evidence for transmission of gut commensals from pets to humans, such as a higher abundance of animal-specific *Bifidobacterium pseudolongum* observed in infants living with pets [35]. Moreover, human microbiomes share more gene content with dog microbiomes than other animals, and humans have a more similar gut microbiome to their own companion animals than they do to companion animals in other households [3, 36]. Immunological priming, or the “hygiene hypothesis,” can be partially explained by pet exposure in that early-life exposure to companion animals can reduce the risk for metabolic and allergic disease [33, 37]. Infants living with household pets were shown to have higher gut diversity than no-pet households [33, 34]. Conversely, young children who developed asthma or allergies were shown to have lower gut diversity than healthy children [38–40], indicating that exposure to pets and resulting “silent” microbial transmission may play important mechanistic roles in the development of human disease. However, our current understanding of why such microbial exposure might improve health outcomes is limited, in part because the gut microbiome within companion animals is uncharted.

Here, we provide the most comprehensive profiling and characterization of the companion animal gut microbiome to date. We collected 2639 dog and cat stool metagenomes, spanning 14 publicly available datasets, and an additional 8 populations provided by Hill’s Pet Nutrition, Inc. (HPN). The metagenomes were analyzed using both reference-based taxonomic and functional profiling, as well as *de novo* assembly for the recovery of novel microbial features. We also incorporated 350 human gut metagenomes for comparison from the Human Microbiome Project 1-II (*n* = 238) [41] and from a Malagasy population (*n* = 112) [42]. We identified taxonomic and functional features that were either host-unique, shared only by companion animals, or shared across companion animals and humans. We also identified patterns of phylogenetic relatedness among strains of microbes observed in the three host species, many of which displayed lineage-specific divergence, whereas others had strains that were phylogenetically similar across hosts (and thus likely frequently transmitted). This study provides a strain-resolved comparison of companion animal and human gut microbiome ecologies, improving our understanding of the transmission of commensals and how microbiome sharing impacts host health.

## Materials and methods

### Study inclusion

We searched PubMed for publicly available, shotgun metagenomic datasets from companion animal hosts published through 2 June 2022. A total of 105 studies were first identified after restricting our search to the English language and combining the following companion animal and microbiology-relevant key terms: “dog,” “cat,” “canine,” “companion animal,” “feline,” “pet,” “gut microbiome,” “gut microbiota,” “metagenome,” “metagenomics,” and “shotgun.” From the set of 105 studies, we (1) reviewed titles, abstracts, and main text to ensure primary research (excluding review manuscripts and non-peer-reviewed research) and excluded studies with (a) samples from animals other than cats and dogs or (b) only 16S rRNA gene amplicons and related non-shotgun sequencing technologies. We then (2) selected for studies with publicly available raw shotgun sequence data, (3) and non-duplicated sample accessions (we encountered this in only one study, in which case the duplicates were not included in our analysis). For one public study [43], samples with duplicated sequencing runs were merged prior to assembly and taxonomic and functional profiling. For another public study [44], 18 of the samples were duplicates, which were subsequently removed after evaluating for correlation with their duplicate pair. We also removed both a sample and its duplicate, as the two were poorly correlated and we were uncertain if this is due to contamination for one or both in the pair. This resulted in 88 samples profiled from the Yarlagadda *et al*. dataset. This resulted in the final dataset [3, 12, 14, 43–53] list provided in Supplemental Table 1.

After screening, raw sequence reads and available metadata were retrieved from the European Nucleotide Archive or Sequence Read Archive. In some cases, metadata were derived from the publications and their supplementary information. Metagenomic samples from the 142 resulting studies were pooled with samples from 87 populations provided by HPN (proprietary) for identical taxonomic and functional profiling through assembly and reference-based methods, followed by downstream phylogenetic and statistical analyses. Human shotgun metagenomes were also retrieved from the Human Microbiome Project 1-II [41] (*n* = 238) and a previously published Malagasy cohort [42] (*n* = 112), bringing the total shotgun metagenomes for analysis to 2989.

### Shotgun metagenomic assembly

To recover metagenomically assembled genomes from the cat and dog gut metagenomes, we carried out quality control and single-sample assembly using the bioBakery workflows [54], with an assembly pipeline previously established for species-level genome bin (SGB) construction [42]. Briefly, we used KneadData v0.7.7 [55] for the quality control of raw sequencing reads (adaptor trimming, removal of low-quality and host reads). The following host genome databases were used for host read removal: GCA_000181335 for cats (*Felis catis*), GCF_014441545 for dogs (*Canis lupus familiaris*), and GRCh37 for humans. Quality-controlled reads were then assembled into contigs using MEGAHIT 1.2.9 (minimum contig length 1500) [56]. Contigs were then binned into metagenomic assembled genomes based on GC content and read depth using MetaBAT v2.15 (minimum contig length 1500) [57]. The assembled genomes were assessed for quality based on completeness (% of expected genes present) and contamination metrics (% of genes with variants or multiple copies of expected single copy genes) using CheckM2 1.0.0 with default parameters [58]. Metagenomic assembled genomes (MAGs) were categorized as high (>90% completeness and <5% contamination), medium (>50% completeness and <10% contamination), or low quality (<50% completeness and >10% contamination). Low-quality MAGs were discarded, and the high- and medium-quality MAGs were placed into species-level genome bins (SGBs) by PhyloPhlAn 3.0 [59] with the “SGB.Jul20” database and default parameters. Each placed MAG was assigned to its reported SGB, genus genome bin, or family genome bin if the Mash distance to the genome bin was <5%, 15%, or 30%, respectively (using previously described thresholds) [42]. MAGs that were not within any of the genome bin thresholds were considered novel, and all novel MAGs across the datasets were clustered into novel SGBs as previously described [42].

### Reference-based profiling with an extended SGB database

The assembled high- and medium-quality genomes were then incorporated as part of an updated MetaPhlAn 4 database [60] of reference genomes and MAGs, and were re-organized into SGBs for marker gene determination (the MAGs from the Coelho *et al*. and Yarlagadda *et al*. datasets were assembled at a later time and will be added to the next MetaPhlAn 4 database update). Ten out of the 19 novel SGBs did not meet the criteria for the minimum number of genomes (≥5) and were not included in the database. With MetaPhlAn 4 v4.0.6 [60] and the updated marker gene database, namely, “Oct22,” we taxonomically profiled the dog, cat, and Human Microbiome Project 1-II [41] samples (where the input was the quality controlled sequencing reads). We also incorporated taxonomic profiles of the gut metagenomes from the Madagascar cohort, for which the raw sequencing reads were processed as previously described [42] and profiled using MetaPhlAn 4 v4.0.64 [60] and the same “Oct22” marker gene database. Three samples from the Madagascar cohort had 100% of reads that were not assigned to any SGB and were therefore removed from the analysis (resulting in 112 samples remaining from 115). SGBs with a relative abundance of at least 10^−5^ in at least three samples from at least one host species were considered, resulting in 2320 SGBs. uSGBs were assigned the closest known taxonomy level and their unique, unchanging SGB number. Taxonomic assignments of SGBs were replaced by those in the “Jun23” marker gene database, which had updated, more accurate taxonomic assignments particularly of uSGBs.

### Phylogenetic analyses

#### Phylogenetic tree of SGBs identified in companion animal gut microbiomes

**Figure 1:**
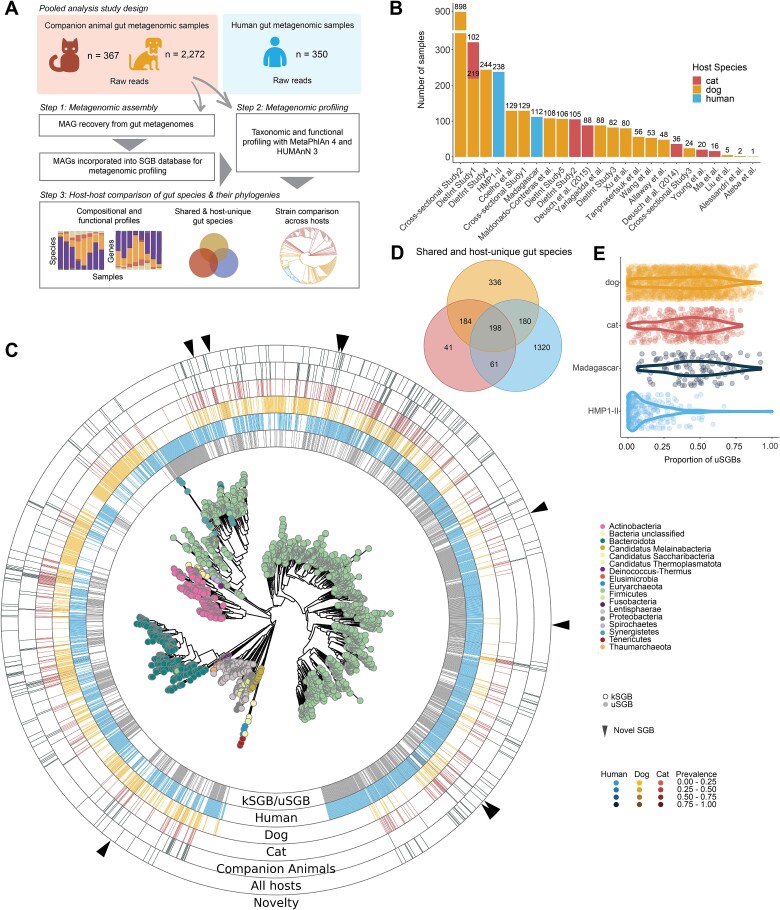
Metagenomic profiling reveals shared and unique SGBs across companion animals and humans. (**A**) Incorporating both reference-free and reference-based methods expands taxonomic profiling of companion animal gut microbiomes. First, MAGs were recovered from companion animal gut metagenomes (*n* = 2639) by single-sample assembly. The resulting genomes were incorporated into an updated MetaPhlAn 4 genome database after SGB clustering. Samples (raw sequence reads) were then taxonomically profiled using MetaPhlAn 4’s marker gene mapping approach (including new SGBs). (**B**) Fourteen public studies and Hill’s Pet Nutrition, Inc. (HPN) contributed varying numbers of dog and/or cat gut metagenomic samples. Two human populations, HMP1-II (baseline samples) (*n* = 238) and a cohort from Madagascar (*n* = 112), were also included in the analysis to compare gut microbiomes across dogs, cats, and humans. “DietInt” means that a particular study contained at least one diet intervention while “NonDietInt” means there was no diet intervention. (**C**) Phylogenetic relationship of the representative genomes for the 2320 SGBs identified with MetaPhlAn 4 (v4.0.6) reveals SGBs that are shared or unique to cats, dogs, and humans. The first ring indicates whether the SGB is known (kSGB) vs. unknown (uSGB), i.e. contained at least one taxonomically assignable genome. The “Human,” “Dog,” and “Cat” rings indicate the prevalence of each SGB in the respective host species. The “Companion Animals” ring indicates SGBs that are shared by cats and dogs but not present in humans. The “All hosts” ring indicates SGBs that were present in cats, dogs, and humans. The triangles on the outer ring refer to novel species recovered from metagenome assemblies of cat and dog gut metagenomes in this study. (**D**) Many SGBs identified in companion animals were host-unique or unique to companion animals and not identified in humans, but an even larger proportion of SGBs identified in humans were human specific. (**E**) A higher proportion of SGBs without confident taxonomic classification (uSGBs) was identified in cats, dogs, and non-Westernized humans compared to Westernized humans. Plot shows the distribution of the proportion of uSGBs (number uSGBs/total number of SGBs) per sample, weighted by relative abundance, across companion animal and human hosts.

The phylogenetic tree (Fig. 1C) was constructed using PhyloPhlAn version 3.0 [59] from representative genomes of the 2320 SGBs identified from taxonomic profiling of the cat, dog, and human gut metagenomes. The representative genomes (highest quality genome for each SGB) were selected using CheckM. The tree was constructed as previously described [60], but instead called on the October 2022 database version with the following parameters: -d phylophlan --diversity high --fast --min_num_markers 50. The resulting tree was visualized using GraPhlAn v1.1.4 [61].

#### Phylogenetic tree of novel SGBs

**Figure 2:**
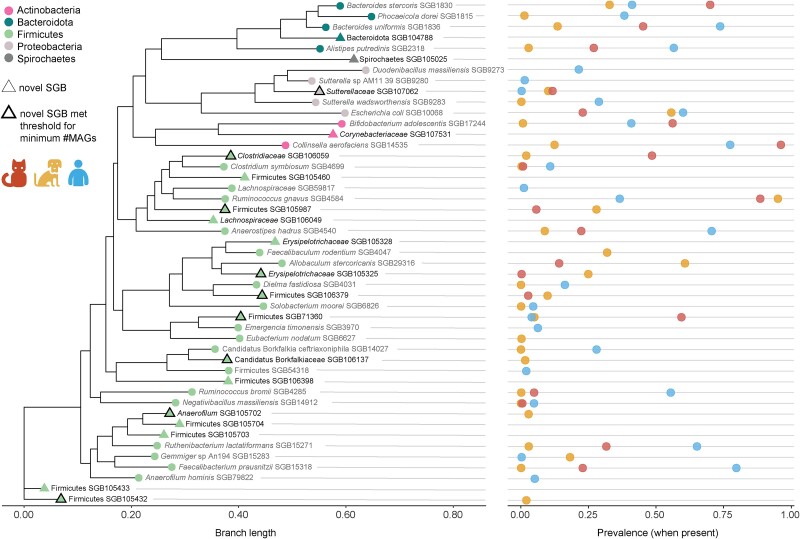
Assembly-based profiling reveals novel SGBs in the companion animal gut microbiome. Nineteen novel SGBs (bold font and denoted by triangles) were identified by metagenomic assembly and span Firmicutes, Bacteroidota, Actinobacteria, Proteobacteria, and Spirochaetes phyla. LEFT: Phylogenetic relationship of the 19 novel SGBs identified from metagenome assembly of the cat and dog gut metagenomes. Nearest kSGBs and other common gut microbes are included for context. RIGHT: Prevalence of SGBs in each host when present (in at least 3 samples) as measured by MetaPhlAn 4. For example, Firmicutes SGB105987 was present in cats and dogs but not present in humans. Ten of the novel SGBs did not meet the minimum number of MAGs per SGB criteria (minimum no. of MAGs = 5) and were not included in the updated MetaPhlAn 4 database (therefore were not identified in taxonomic profiles using MetaPhlAn 4 and thus are missing prevalence information).

To place the 19 novel SGBs identified from this dataset into a phylogenetic context, we built a phylogenetic tree from genomes representative of these novel SGBs, their nearest kSGB neighbors from the phylogenetic tree in Fig. 1C, and several additional common gut microbes (Fig. 2). The tree was constructed using PhyloPhlAn version 3.0 and the following parameters: -d phylophlan --diversity high --fast. The resulting tree was visualized using the ggtree v3.2.1 R package. This phylogenetic tree does not include data from Coelho *et al*. and Yarlagadda *et al*. because they were added to the analysis at a later time, as mentioned above.

#### Phylogenetic structure of individual SGBs

We evaluated the patterns of strain similarity found across SGBs common to host species. A multiple sequence alignment of consensus strains identified for each SGB by StrainPhlAn 4 [60] was used to build phylogenetic trees for individual SGBs. Trees were built using the ggtree v3.2.1 R package and strains (tree tips) were colored to note host origin.

### Calculating beta-diversity of taxonomic profiles

Bray–Curtis dissimilarities of taxonomic profiles were calculated using the R vegan v2.6-4 package (https://github.com/vegandevs/vegan) and visualized using a principal coordinate analysis (PCoA) with the “ggplot2” R package. Due to the disparity in sample size across host species, the principal component scores (PCs) were frequency corrected by their respective host-specific sample sizes. For instance, the PC scores corresponding to samples from cats were corrected by dividing the scores by 367, the total number of gut metagenomic samples from cats.

**Figure 3:**
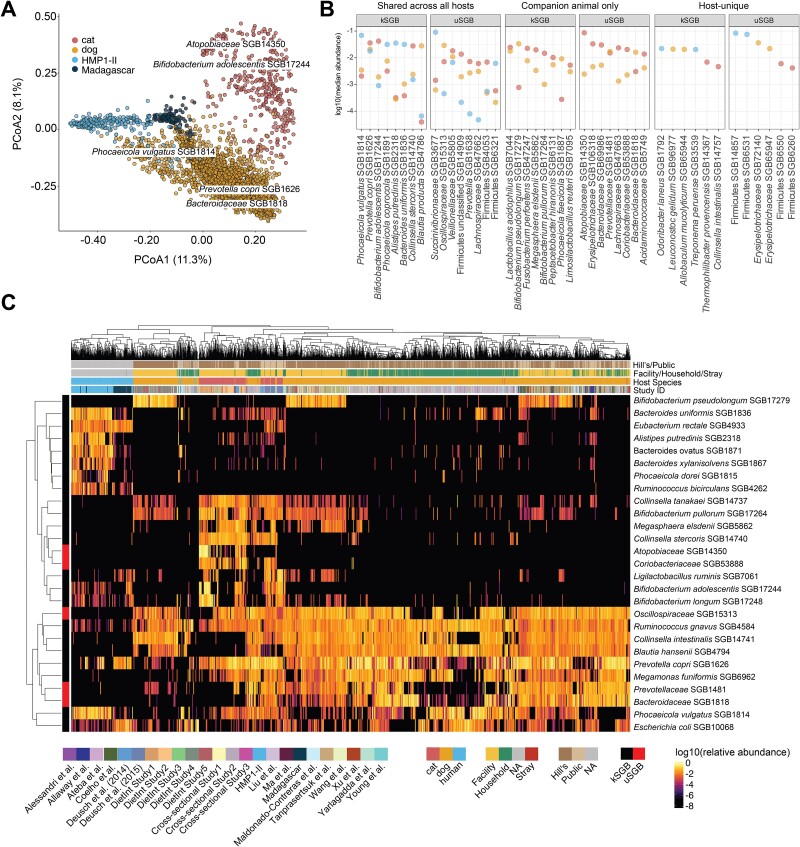
Taxonomic profiles are distinct across host species. (**A**) Host species is the dominant determinant of microbiome structure, as expected. Frequency-corrected principal coordinates analysis (PCoA) (by Bray–Curtis dissimilarity) indicated 4 major clusters of gut communities (PERMANOVA *R*^2^ = 0.03, *P* value = .001). PC scores were corrected by dividing the scores by the sample sizes of the respective host species. Major taxonomic drivers of the clustering are annotated and their positions are determined by the weighted average PC scores. (**B**) Subsets of individual SGBs follow all combinations of sharing patterns among companion animals and humans, sometimes at high abundance. We identified 198 SGBs that were shared across cats, dogs, and humans, 184 SGBs shared between companion animals which were not present in the human gut metagenomic samples (HMP1-II and Madagascar cohorts), and 1697 SGBs unique to one of the three hosts (cat = 41, dog = 336, and human = 1320 unique SGBs). Panels show the most abundant kSGBs and uSGBs in each category. Values on the *y*-axis denote median relative abundance when present. (**C**) Taxonomic profiles clustered based on the most abundant gut microbes (the 27 gut species are the union of 10 most abundant microbes per host species). See **Methods** for filtering and selection criteria. Both columns (SGBs) and rows (samples) are hierarchically clustered using Euclidean distances. Column annotations indicate key metadata features including data source type, housing condition, host species, and study ID. Row annotations denote whether the SGB is known or unknown. “DietInt” refers to studies with dietary interventions.

To compare bacterial composition across host species and other metadata such as housing and study information, we applied hierarchical clustering to the taxonomic profiles and plotted the relative abundance of the most abundant species by sample (R ComplexHeatmap [62] v2.13.1 package). We applied a prevalence and abundance filter, by host species, to capture gut species with a relative abundance of at least 1 × 10^−5^ in at least 25% or 10% of samples in at least two studies. The resulting subset of gut species (Fig. 3B) is the union of the 10 most abundant gut species identified in each host species. SGBs with relative abundance >1 × 10^−5^ in at least three samples within one host species were considered present when calculating prevalence.

### Statistical analysis of taxonomic profiles

Permutational multivariate analysis of variance (PERMANOVA) was performed to analyze the association between microbial communities and “study” ID, “host species,” housing, and age (univariately) using the adonis2 function in the vegan v2.6-6.14 R package. The model is as follows: adonis2(abundance matrix ~ study + host species, by = “margin”, permutations = 999, method = “bray”). Each univariate analysis was based on the complete cases of the available sample metadata. We did not include the terms “housing” or “age,” as housing information/variation exists predominantly for dogs, and age (measured in years) does not translate to the same life stage across hosts. We also tested whether housing differences drive variation in the gut microbiome of dogs using the following model in adonis2: adonis2(abundance matrix ~ age + housing, by = “margin”, permutations = 999, method = “bray”). We did not include “study” as a variable in this model as study and housing are confounding variables. This would provide a maximum “ceiling” estimation of the variation in taxonomic community due to differences in housing. Samples from stray dogs (*n* = 14) were not included in this model due to the imbalance of group size.

To identify gut bacterial species associated with host species and housing type, we applied a linear mixed effect model (R MaAsLin 2 v1.8.0 package) [63]. The model was run with MaAsLin’s default settings, where feature tables are normalized by total sum scaling and log-transformed. Bacterial taxa with relative abundance >10^−5^ in at least 20 samples in at least one host were included in this analysis. We first identified gut species associated with host species using MaAsLin 2, specifying host species and housing (facility vs. private household vs. stray) as fixed effects, study ID and subject as a random effects, and dogs as the reference variable for the host species fixed effect (and facility as reference for the housing variable). To carry out all comparisons between cats, dogs, and humans equivalently, we repeated the same model but instead specified cats as the reference variable for host species. The effect sizes and *P* values from both iterations were combined and the *P* values were corrected for the false discovery rate (FDR) using the Benjamini and Hochberg correction method. We next examined the association between gut species and housing (facility vs. private household) within dog samples only, specifying the study ID as a random effect. Cats were excluded in this model because all cats were facility housed.

### Phylogenetic coherence score among hosts

The coherence score, previously described as the niche-association score [41, 64], determines the niche specificity of phylogenetic subclades. In our case, niche refers to the uniformity of host species within strain lineages, as we sought to identify SGBs with subclades associated with different hosts (cats, dogs, and HMP1-II human samples). First, Kimura 2-parameter distances were calculated from the multiple sequence alignment produced by StrainPhlAn 4 using the “distmat” function as part of the EMBOSS v6.6.06.4.0 [65] software package. For SGBs identified in more than one host (see criteria below), a host-specific score is first calculated, which compares the genetic distances of strains from one host compared to the genetic distances of strains from the other host(s), as previously described [41, 64]. The final coherence score assigned to an SGB was calculated using the mean of the host-specific scores. In order for a coherence score to be calculated, the SGBs were required to be (1) shared (i.e. SGBs unique to one host species were excluded), (2) present in at least 20 samples with sufficient coverage as determined by StrainPhlAn 4, and (3) present in at least 5 samples from at least 2 hosts.

### Functional profiling of companion animal and human metagenomic profiles

Metagenomes were functionally profiled using HUMAnN v3.6 [55] (using bioBakery [55] v3.0.0-beta, including MetaPhlAn v3.0.14 taxonomic profiles). HUMAnN generated a microbial species-stratified gene family table, which was annotated at the UniRef90 level. The HUMAnN utility script humann_split_stratified_table created an unstratified version of the gene family table, which was used in the antimicrobial resistance (AMR) analysis. Functional profiles from the Madagascar cohort were excluded from downstream functional analysis due to poor coverage.

### Strain phylogeny and gene carriage statistics using Anpan

To evaluate within-microbe gene carriage differences across hosts, we used the “gene model” implemented in the software package Anpan v0.3.0 (Analyses of microbial phylogenies and genes; https://huttenhower.sph.harvard.edu/anpan). The gene model takes as input the microbial species-stratified gene family table (generated by HUMAnN) and finds within-species genes associated with an outcome (outcome refers to host, in our case), first by filtering for genes that are well covered across samples, followed by running a logistic regression model with an FDR correction to identify genes significantly enriched in a given host. The model was applied pairwise for three sets of hosts: cat versus dog, cat versus human, and dog versus human. The command anpan_batch() was called and recursively runs the gene model for multiple microbial species. The following parameters were specified: filtering_method = “kmeans”, model_type = “fastglm”, omit_na = “TRUE”, and outcome = “host_species”, beta_threshold = 1.25, q_threshold = 0.1.

We calculated the number of differential genes between pairs of hosts as identified by Anpan (absolute effect size >1, *q* < 0.1). The edge values are stratified by the number of genes enriched in each host of the pair. The number of genes enriched in each host were defined as the number of the union of genes significantly enriched in host X versus host Y and host X versus Z. Values were normalized by the species’ pangenome size and are presented as the number of genes per 1000 genes (UniRef90s). To visualize the most differential genes across hosts in *Ruminococcus gnavus*, we selected the union of the 20 genes from each pairwise test that had the largest absolute effect size with a *q*-value <0.1 and plotted presence/absence of these genes in a heatmap (R ComplexHeatmap v2.10.0 package). The filtered_Ruminococcus_gnavus.tsv files generated from the filtering step for each of the three pairwise runs were used as input for the heatmap.

### AMR gene mapping and analysis of ARGs

To identify genes associated with antibiotic resistance, we aligned query protein sequences from The Comprehensive Antibiotic Resistance Database v.3.2.7 (CARD, https://card.mcmaster.ca) [66, 67] with protein sequences from the uniref90_annotated_v201901b_full database using diamond v2.0.4 [68] using the following command: diamond blastp -d uniref90_201901.dmnd -q protein_fasta_protein_homolog_model.fasta --outfmt 6 qseqid sseqid qlen qstart qend slen sstart send length bitscore evalue pident qcovhsp scovhsp --out output.tsv. Alignments with at least 90% identity and 80% mutual coverage were selected for the analysis, resulting in 598 UniRef90 (CARD-annotated) gene families that are referred to in the manuscript as antibiotic resistance genes (ARGs). From this subset, gene families were further filtered to keep only those present (abundance >0) in at least three samples in at least one host. This final set of 396 ARGs was evaluated to understand how AMR features are distributed across companion animals and humans. The variation in ARG profiles across hosts was quantified using a univariate PERMANOVA (adonis function in R vegan package v2.6-4). Based on the CARD annotations, we classified ARGs into groups of antibiotics to which the genes confer resistance (the abundance of the ARGs in each antibiotic group, for each host, was summed). We applied MaAsLin 2 v1.8.0 to quantify differences in the carriage of ARGs across hosts (either for the ARGs individually or for the aforementioned groups of ARGs conferring resistance to the same antibiotics).

## Results

### Reconstruction of metagenomically assembled genomes from companion animal microbiomes reveals 19 novel species and expands reference-based profiling

We profiled a total of 2639 shotgun-sequenced cat and dog gut metagenomes from 14 publicly available datasets and newly sequenced metagenomes from HPN (Supplemental Table 1, Fig. 1A–B, see **Methods**). These included 367 cat metagenomes (from 133 cats) sourced from 4 public studies and HPN, and 2272 dog metagenomes (from 1378 dogs) sourced from 10 public studies and HPN (Fig. 1B). The cat and dog stool samples spanned early life to old age (5.57 ± 4.77 and 8.10 ± 3.72 years for cats and dogs, respectively), a range of weights (5.29 ± 0.99 and 15.99 ± 13.17 kg for cats and dogs, respectively), healthy and disease phenotypes (e.g. chronic enteropathy, obesity), dietary interventions, antibiotic and other medication use, and facility versus household residence (Supplemental Fig. 1, Supplemental Table 1**,**Supplemental Table 15). To compare the gut microbiome features of cats and dogs to humans, we incorporated 350 gut metagenomes from two human populations, one Westernized (first collected “baseline” samples from the HMP1-II cohort [41]) (*n* = 238) and one non-Westernized population from Madagascar (*n* = 112) [42] (Fig. 1B). We included both Westernized and non-Westernized human populations to encompass humans that (1) shared similar environments to the animals sampled in this study, and (2) would have had little related contact or shared environment. We performed a methods-standardized pooled analysis that taxonomically and functionally profiled gut metagenomes from cat, dog, and human hosts using reference-free (cat and dog samples) and reference-based (all samples) methods, followed by a strain-level phylogenetic comparison of the microbes identified across hosts (Fig. 1A).

As the gut communities of cats and dogs are relatively underexplored, we first applied single-sample assembly followed by species-level genome binning (SGB, **Methods**) to identify novel clades. From the 2639 cat and dog gut metagenomes, this recovered a total of 61 515 MAGs. After quality control [42], 7275 high-quality and 21 706 medium-quality MAGs (Supplemental Fig. 2, **Methods**) were incorporated as part of an updated MetaPhlAn 4 database [60] after grouping the MAGS into SGBs for marker gene determination. Notably, incorporation of these MAGs into the database increased the number of reads that could be used for taxonomic classification and, thereby, the estimated “known” proportion of communities as calculated by MetaPhlAn (Supplemental Fig. 3). In other words, this improved both specificity and, especially, sensitivity of taxonomic profiling for companion animal gut metagenomes.

Using the resulting updated MetaPhlAn 4 database, we taxonomically profiled the 2989 companion animal and human metagenomes, which detected 2320 SGBs. These predominantly spanned Firmicutes (66%), Bacteroidota (formerly known as Bacteroidetes) (11%), Proteobacteria (8%), and Actinobacteria (9%), as expected (Fig. 1C, Supplemental Tables 2–4). Next, we identified SGBs that were either host-unique (#SGBs in cats = 41, dogs = 336, humans = 1320), in cats and dogs only (#SGBs = 184), in humans and cats (#SGBs = 61), in humans and dogs (#SGBs = 180), or shared across all three hosts (#SGBs = 198) (Fig. 1D). SGBs containing at least one characterized isolate genome in the database are called known SGBs (kSGBs) (*n* = 1041 in our dataset), whereas those lacking characterized isolate genomes are unannotated at the species (or higher) level and are referred to as unknown SGBs (uSGBs) (*n* = 1279). The proportion of SGBs lacking confident taxonomic classification (i.e. uSGBs) in companion animals and non-Westernized humans was greater than in Westernized humans, by virtue of having relatively uncharacterized gut microbiomes from which fewer isolates have been derived (Fig. 1E).

By assembling new genomes, we identified 19 novel SGBs in the cat and dog metagenomes that span Firmicutes, Bacteroidota, Actinobacteria, Proteobacteria, and Spirochaetes (Fig. 2). Of the 19 novel SGBs, 9 were sufficiently prevalent (**Methods**) to be represented in the marker gene database for taxonomic profiling by MetaPhlAn 4. Three of these were found only in dogs (Firmicutes SGB105432, *Anaerofilum* SGB105702, and *Candidatus Borkfalkiaceae* SGB106137) and four were found in both cats and dogs (Firmicutes SGB106379 and SGB105987, *Erysipelotrichaceae* SGB105325, and *Clostridiaceae* SGB106059). The remaining two novel SGBs recovered from cat and dog metagenomes (*Sutterellaceae* SGB107062 and Firmicutes SGB71360) were also present in humans, albeit at low prevalence (<1% and 4%, respectively).

### Most microbial taxa are host specific among companion animals and humans

At the community level, we identified significant clustering by host and study (PERMANOVA *R*^2^ = 0.03, *P* value = .001; *R*^2^ = 0.22, *P* value = .001, respectively) (Fig. 3A, **Methods**). An evaluation of the microbial communities using a phylogeny-aware distance method (i.e. UniFrac) indicated more overlap between the hosts’ gut communities, accounting for phylogenetic similarity in microbial lineages (Supplemental Fig. 4). The most abundant microbial species in each host showed differential carriage patterns; only three species overlapped as being within the top 10 abundant species per host (Fig. 3C). Additionally, overall community composition also differed within hosts. For example, human gut communities were easily distinguishable between Westernized (HMP1-II) and non-Westernized (Madagascar) populations (Fig. 3A,C), as seen before [42]. Within dogs, gut communities clustered based on housing, i.e. facility versus private households (PERMANOVA *R*^2^ = 0.12, *P* value = .001) (Fig. 3C, Supplemental Fig. 5, Supplemental Table 8). Compared to dogs housed in facilities and private households, stray dogs were enriched in *Helicobacter bilis* SGB19390, which was virtually absent in facility-housed dogs (β = 10.71, *q* < 0.001). Conversely, the microbe *Turicibacter sanguinis* SGB6846 was absent in stray dogs but prevalent in both private and facility-housed dogs (Supplemental Fig. 5). Compared to dogs in private households, facility-housed dogs were enriched in Firmicutes SGB105987 (one of the novel SGBs identified in this dataset) (β = 2.57, *q* = 0.010), *B. pseudolongum* SGB17279 (a commensal in dogs, β = 5.39, *q* < 0.001), along with other unclassified SGBs including *Collinsella* SGB14744 (β = 2.55, *q* = 0.035) and *Lactobacillaceae* SGB7083 (β = 4.88, *q* < 0.001). Contrastingly, *Tyzzerella nexilis* SGB4588, *Bilophila wadsworthia* SGB15452, and *Succinivibrionaceae* SGB3675 were among microbes with the largest effects sizes that were found in household dogs but absent in facility-housed dogs (β = 2.46; β = 0.29, and 0.287, respectively, all with *q* < 0.050). Both *T. nexilis* and *B. wadsworthia* are human gut commensals and their alterations (changes to abundance and metabolism) have been previously implicated in type 2 diabetes and inflammatory bowel disease (IBD) in both humans and mice [69, 70]. In part owing to other variables such as differences in diet, exercise, and environment, the presence of these microbes in dogs dwelling with humans (but absent in dogs with arguably less human interaction) suggests sharing of, in this case, pathobiont gut microbes between humans and pets.

### Niche specificity of gut microbes identified across cats, dogs, and humans

Several well-characterized human gut microbes, including *Phocaeicola vulgatus* SGB1814, *R. gnavus* SGB4584, and *Prevotella copri* SGB1626, were shared among all hosts (Fig. 3B–C, Supplemental Fig. 6, Supplemental Tables 5 and 8). Many shared microbes were differentially abundant across hosts, potentially indicating diverged ecotypes or strains associated with different host physiologies, diets, and environments. For example, despite the universality of *Bifidobacterium adolescentis* SGB17244, its abundance was higher in cats than in dogs (β = 9.17, *q* < 0.001) or humans (β = 6.64, *q* = 0.002). Members of the *Collinsella* genus, including *Collinsella stercoris* SGB14740, *Collinsella tanakaei* SGB14737, and *Collinsella intestinalis* SGB14741, were almost absent in humans (2%–5% prevalence), but were present in at least 25% of cats and dogs (with the exception of *C. stercoris* SGB14740, which was found in 8% of dogs). Intriguingly, *R. gnavus* SGB4584, a microbe that is often disease associated in humans [71] [72], was also more abundant in dogs than in cats and humans (β = 3.71, *q* < 0.001; β = 10.40, *q* = 0.001, respectively) (Fig. 3C, Supplemental Fig. 6), suggesting a different ecological niche preference for *R. gnavus* in canines (analogous to e.g. *Helicobacter* in nonhuman primates [73]).

Companion animal–specific microbes included species belonging to *Bifidobacterium* (*Bifidobacterium pullorum* SGB17264 and *B. pseudolongum* SGB17279), *Lachnospiraceae* SGB4589, *Megasphaera* (*Megasphaera elsdenii* SGB5862 and *Megasphaera stantonii* SGB5854), *Collinsella* (e.g. *Collinsella phocaeensis* SGB109036), *Prevotellaceae* SGB1481, and *Peptacetobacter hiranonis* [6, 74] SGB6131, to name a few. Among the most prevalent SGBs shared only by cats and dogs were the unclassified *Blautia* SGB4793, *Lachnospiraceae* SGB4870, Firmicutes SGB4668, *Atopobiaceae* SGB14350, and *Lachnospiraceae* SGB4859. Some of these uSGBs were very prevalent (e.g. *Blautia* SGB4793 was observed in 97% of cats), underscoring the need for characterization of animal-specific gut microbiomes (Supplemental Tables 5 and 8).

Among the most prevalent (>30%) SGBs unique to dogs were *Turicibacter* sp. 1E2 SGB39153, *Erysipelotrichaceae* SGB42251, *Faecalibaculum rodentium* SGB4047, and *Bifidobacterium canis* SGB53847. The most prevalent cat-unique SGBs were Firmicutes bacterium AM41_11 SGB6817, *Faecalicoccus pleomorphus* SGB6791, Actinobacteria SGBs 13825 and 53822, *Enorma burkinafasonensis* SGB86218, *Olsenella timonensis* SGB14360, and several *Megaspaera* spp. (SGBs 5852, 5861, 5863) (Supplemental Tables 5 and 8). Certain *Slackia* spp. were unique to either cats or dogs (*Slackia equolifaciens* SGB33546 in cats; *Slackia faecicanis* SGB33547 and *Slackia* SGB14780 in dogs). Many were rare, like Firmicutes SGB6260, which was present in 5.2% of cats but absent in humans and dogs. *Roseburia hominis* and *Faecalibacterium prausnitzii* SGBs were either unique to or much more prevalent in humans, respectively. *Faecalibacterium prausnitzii* SGBs were present in ≤0.3% of dogs, up to 23% of cats, and up to 85% in humans. These species are well-studied human gut residents with protective anti-inflammatory properties [75, 76]. Two *C. tanakaei* SGBs and two *C. intestinalis* SGBs were identified in cats, whereas only one of each was found in dogs and only 2% and 4% of humans, respectively. These findings indicate host-unique selective pressures on community assemblage, leading to niche specificity (i.e. host preference) even at the subspecies level.

We also identified microbes known for zoonotic transmission, including *Helicobacter* and *Campylobacter*. *Helicobacter* spp. (non–*Helicobacter pylori* species) are commonly found in the gastrointestinal tract of companion and other animals and are known to cause human and animal gastritis [77–82]. Common to cats and dogs were *Helicobacter canis* SGB19391, *Helicobacter cinaedi* SGB44267, *Helicobacter winghamensis* SGB19399, *H. bilis* SGB19390, and the unclassified *Helicobacter* SGBs 28474 and 104937 (Supplemental Table 6). A different *H. canis* subclade (i.e. SGB21969) was found only in dogs, indicating a niche preference for subspecies clades in a zoonotic-relevant microbial species. The *Helicobacter* spp. that we identified are consistent with those identified in cats and dogs in previous studies [83–85]. The human pathogen *H. pylori* was found only in two human samples as previously described [86], and was not detected in cats and dogs, as expected [87]. Inconsistent with previous studies was the absence of *Helicobacter heilmannii* and *Helicobacter suis* in the current dataset [82, 83, 85]. *Campylobacter*, which is commonly carried in companion animals and implicated in human and dog enteritis [88, 89], was observed but not as prevalent as previously reported [90, 91]. *Campylobacter jejuni* SGB19444 was present at low prevalence in dogs and humans (Supplemental Table 7). The remaining *Campylobacter* spp. were either companion animal specific (*Campylobacter upsaliensis*, *Campylobacter helveticus*, *Campylobacter coli* SGB19443, and the unclassified *Campylobacter* SGB19337) or human specific (*Campylobacter concisus* SGB19351, *Campylobacter hominis* SGB19429). More work is needed to understand whether there are shared or unique virulence factors across host-specific SGBs, especially those belonging to the same microbial species and differing only in strain genetics.

### Lineage-specific divergence within gut species shared among hosts

**Figure 4:**
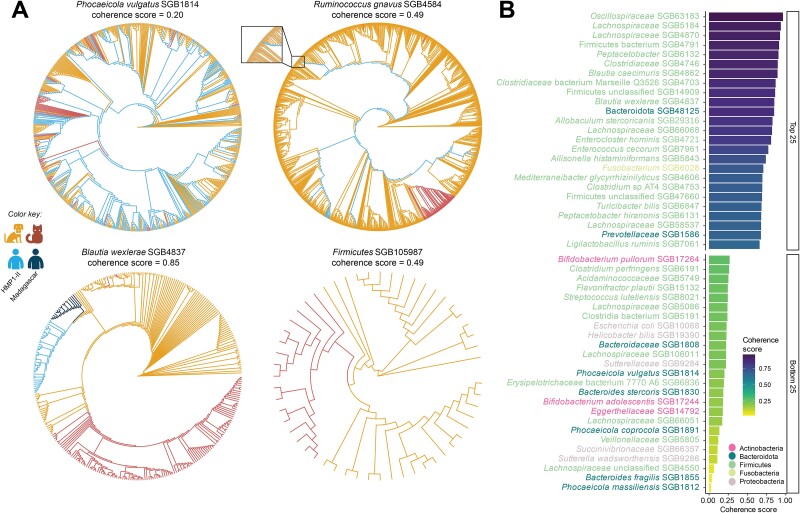
Lineage-specific divergence across gut microbes. (**A**) We identified significantly different phylogenetic distributions of subspecies clades among hosts for several SGBs. Strains of *Ruminococcus gnavus* SGB4584 distinctly cluster by host species. Occasionally, single human or cat-recovered strains cluster with strains recovered from dogs, suggesting possible recent transmission events (inset). In cases like *Phocaeicola vulgatus* SGB1814, strains are universally similar across hosts, indicating frequent transmission. Phylogenetic trees were built from multiple sequence alignment using StrainPhlAn 4. (**B**) Across SGBs, the genetic similarity of strains varied across hosts. This is quantified by the coherence score, where larger values indicate greater divergence of subspecies clades across host species. Larger values thus indicate greater divergence and less frequent transmission among hosts. The coherence score relies on Kimura 2-parameter distances calculated from a multiple sequence alignment of consensus strains identified for each sample by StrainPhlAn 4 (see **Methods**).

On account of the observed niche specificity of shared SGBs, we wanted to understand how subclades compare phylogenetically across hosts. We first qualitatively observed both lineage-specific divergence and strain similarity among SGB subclades across different hosts (Fig. 4A and Supplemental Fig. 7). For instance, *R. gnavus* SGB4584, *Blautia wexlerae* SGB4837, and Firmicutes SGB105987 (one of the novel SGBs identified in this dataset) all displayed lineage-specific genetic divergence, where strains from the same host formed clear subclades within the SGB’s phylogenetic tree. In contrast, strains of *Phocaeicola vulgatus* SGB1814 and *Bacteroides stercoris* SGB1830 were genetically similar across hosts (Fig. 4A and Supplemental Figs 7–8). This suggests either a higher rate of transmissibility between hosts or (unlikely) convergent evolution of strains, most likely due to similar functional landscapes across host species.

We next calculated a coherence score to quantify these different patterns of divergence and (likely) transmissibility [41, 64]. Specifically, the coherence score measures the distinctness of subclades based on host species by comparing the genetic distances of strains identified in a host to those in other host(s); a higher coherence score thus indicates higher genetic divergence among hosts and less frequent putative transmission (Fig. 4B and Supplemental Fig. 9, **Methods**). For species shared by at least two hosts, we observed a wide range of coherence scores, indicating that some microbial species maintain host-specific lineages, whereas others exhibit more frequent gene flow. Among SGBs with the highest divergence across host species were members of Firmicutes, including the uSGB *Lachnospiraceae* SGB4870 (with the highest mean coherence score of 0.924), *Blautia caecimuris* SGB4862, and *Blautia wexlerae* SGB4837 (mean coherence scores 0.892 and 0.850, respectively). This phylum, and other high-scoring clades, were thus most likely to be host specific. In contrast, most Actinobacteria and Bacteroidota displayed modest host-based clustering.

Some species showed divergence across all hosts, whereas others had a subclade associated with one host but were similar across the other two. For instance, *B. wexlerae* SGB4837 displayed lineage-specific divergence for all three hosts (host-specific coherence scores of 0.763, 0.876, and 0.909 for human, cat, and dog clusters, respectively, with a mean coherence score = 0.850), whereas *B. stercoris* SGB1830 and Clostridia bacterium SGB5191 each had only a cat-specific subclade (*B. stercoris* SGB1830: host-specific coherence scores of 0.00, 0.554, and 0.00 for human, cat, and dog clusters, respectively, with a mean coherence score = 0.185; Clostridia bacterium SGB5191: host-specific coherence scores of 0.00, 0.623, and 0.093 for human, cat, and dog clusters, respectively, with a mean coherence score = 0.238) (Fig. 4B and Supplemental Figs 8–9).

We also observed cases where SGBs demonstrated genetic similarity across hosts, i.e. putatively more frequent transmission events. When shared, members of Bacteroidetes and especially the genus *Phocaeicola* tended to show this behavior (Fig. 4B and Supplemental Fig. 9). Other selected species with strain similarity across hosts included *P. massiliensis* SGB1812 (mean coherence score = 0.024), *B. fragilis* SGB1855 (mean coherence score = 0.036), *P. copri* SGB1626 (mean coherence score = 0.509), *Veillonellaceae* SGB5805 (mean coherence score = 0.119), and *Sutterellaceae* SGB9284 (mean coherence score = 0.220), among others (Fig. 4A–B**,** and Supplemental Figs 8–9).

There were differences in host-lineage specificity even in common gut commensals of companion animals such as *Bifidobacterium* (Fig. 4B and Supplemental Fig. 9). We found that *Bifidobacterium longum* SGB17248, e.g., had less genetic similarity across hosts relative to *B. pseudocantenulatum*. In a previous study, the relatedness of *Bifidobacterium* strains isolated from non-cohabiting cats, dogs, and humans varied between *Bifidobacterium* species, with less genetic similarity of strains across hosts in *B. pseudolongum* and *Bifidobacterium pseudocatenulatum* compared to *B. longum*. However, that evaluation was made only with isolates from a small number of cats and dogs [92].

### Strain-level functional variation suggests adaptation to host environment

**Figure 5:**
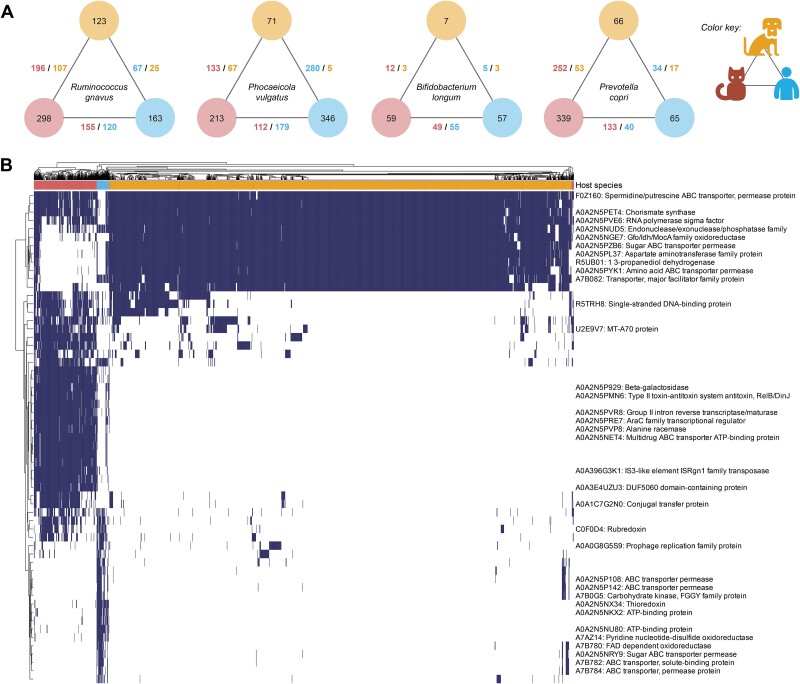
Differences in strain-level gene carriage suggest adaptation to different host environments. (**A**) Differential gene carriage in host-specific strains of shared microbes. For each microbial species, edge values refer to the number of significantly enriched genes in each host for that respective pairwise comparison (number of significantly enriched genes in pairwise tests are noted for cats, dogs, and humans) (Anpan |effect size| ≥ 1.0, *q*s ≤ 0.10) (**Methods**). Node values signify the total number of significantly enriched genes carried by the respective microbe found in each host (the total number of genes unique to a host across pairwise host comparisons) (**Methods**). Values were normalized by the species’ pangenome size and are presented as the number of genes per 1000 genes (UniRef90s). (**B**) The functional capacity of *R. gnavus* is host specific. The heatmap shows the presence/absence of the most differential genes (rows, genes are annotated by the UniRef90 ID) measured for a pair of hosts, i.e. the 20 genes with the largest absolute value of the effect size for significantly different genes (*q* < 0.1) in each pairwise host comparison. Missing UniRef90 IDs denote gene families that do not have a functional annotation in the HUMAnN 3.6 database.

To understand the functional implications of these phylogenetic differences across subspecies, we applied a logistic regression model to identify within-species gene carriage associated with hosts (pairwise host comparisons, see **Methods**). Some species were again more diverged across hosts than others, i.e. had higher variation in gene carriage between hosts (Fig. 5A and Supplemental Fig. 10). For example, there were hundreds of genes that differed between *R. gnavus* found in different hosts, whereas *B. longum* gene carriage differed between hosts by as little as one gene or by no genes at all. This emphasizes the difference in phylogenetic divergence versus gene carriage, the former of which can occur either because of gene gains/losses or from polymorphisms independent of structural variation. Depending on the species, strains were often more similar in gene carriage among companion animals (cats vs. dogs) (i.e. *B. longum*, *Bacteroides vulgatus*, and *B. stercoris*). In contrast, *P. copri* and *R. gnavus* strains found in dogs and humans were more similar in gene carriage patterns.

We were particularly interested in host-specific gene carriage of *R. gnavus*, which is core to the companion animal gut microbiome and disease relevant in both animals and humans [6, 71, 93]. *Ruminococcus gnavus* displayed distinctive gene carriage profiles that suggest, at the least, adaptation to nutritional requirements across hosts (Fig. 5B, Supplemental Figs 11–12). For instance, cats are obligate carnivores and consume far less fiber than dogs and especially humans. Certain amino acid synthesis genes were enriched in companion animal *R. gnavus* strains compared to humans, such as the gene synthesizing chorismate (UniRef90 A0A2N5PET4), a precursor to several aromatic amino acids including tyrosine (essential to cats only) [94–96]. Dogs were conversely enriched in the *artM* gene, which belongs to an arginine transport system [96] (Supplemental Fig. 11). Although these nutrient-based differences are less likely to be drivers of disease phenotypes, as compared to the many uncharacterized genes also differential among strains, they do suggest ways in which microbial lineages maintain host specificity.

*Ruminococcus gnavus* strains also diverged in carbohydrate metabolic functions, likely due to differences in host carbohydrate consumption (cats again consume less than dogs and humans) [97, 98] (Fig. 5B and Supplemental Figs 11–12). These included a beta-galactosidase (cats), aspartate aminotransferase (dogs), *araC*—which regulates the transcription of arabinose metabolism enzyme genes (different gene families from the AraC family of transcriptional regulators were found in cats vs. dogs)—and the FGGY family of carbohydrate kinases (humans) (*q*s < 0.10). *Ruminococcus gnavus* in cats and humans shared the same oxaloacetate decarboxylase (UniRef90 A0A2N5P6J1), whereas a different oxaloacetate decarboxylase gene family (UniRef90 A0A2N5PES0) was unique to dogs.

The hosts further carried distinct glycosidase and glycotransferase profiles (Supplemental Fig. 13). Glycosidases are known to vary across mammalian hosts [99], which is indicative of niche adaptation to different diets and/or physiologies (e.g. mucus composition). *Ruminococcus gnavus* strains were previously shown to have distinct glycosidases specific to human blood groups (*R. gnavus* degrades mucin and different strains have substrate preferences for mucin-type *o*-glycans) [100, 101]. As cats, dogs, and humans have different blood antigens [102], differential carriage of glycosidases further suggests host specificity as a result of differences in carbohydrate (glycan) availability. These differences in gene carriage highlight the adaptability of the microbiota to different host environments and response to dietary intake.

### Host divergence in ARG profiles

**Figure 6:**
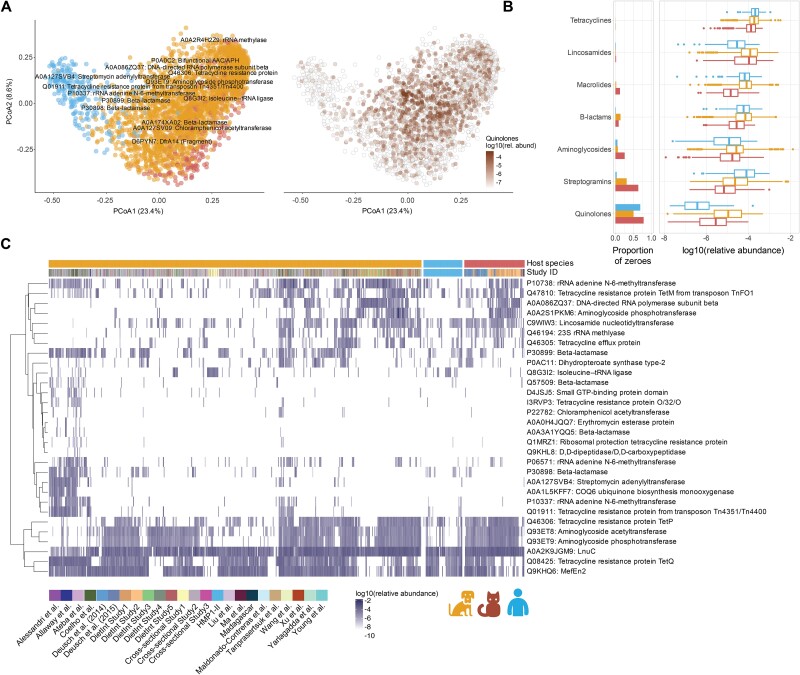
Companion animals harbor antibiotic resistance genes active against host-specific drug targets. (**A**) Host species drive the variation in beta-diversity of ARG profiles. Frequency-corrected principal coordinates analysis (PCoA) (by Bray–Curtis dissimilarity) shows distance of ARG profiles derived from companion animal and human gut microbiomes (PERMANOVA *R*^2^ = 0.11, *P* value = .001). Samples are colored by host species or relative abundance of genes conferring resistance to quinolones. PC scores were corrected by dividing the scores by the sample sizes of the respective host species. Major drivers of the clustering are annotated and their positions determined by the weighted average PC scores. (**B**) Resistance to tetracyclines, lincosamides, macrolides, β-lactams, and aminoglycosides were universal and abundant across all three hosts relative to other antibiotic resistance factors. The total abundance of ARGs conferring resistance to each antibiotic was compared, see **Methods.** Values are relative abundance. (**C**) ARGs were differentially abundant across cats, dogs, and humans. Heatmap shows the union of the 15 most differential ARGs per host (based on MaAsLin 2 effect sizes, qs < 0.25). “DietInt” refers to diet intervention studies.

Among functional differences among host-specific microbes and gene carriage, AMR is perhaps best characterized and well detectable. ARGs can also be under extremely high selective pressure, and transmission of resistant bacteria can occur between pets and their cohabitating pet owners [103–107]. To assess the diversity of ARGs across hosts, we compared the ARG profiles derived from the hosts’ metagenomes by mapping the gene families to the CARD database [108] (**Methods**). We observed significant variation in profiles between different hosts (PERMANOVA *R*^2^ = 0.11, *P* value = .001) (Fig. 6A,C). ARGs were detected conferring resistance to several antibiotic classes, ranging from those commonly prescribed in small animal veterinary medicine (e.g. β-lactams, macrolides, tetracyclines) to those approved for livestock or humans only (e.g. pleuromutilins) (the total abundance of ARGs conferring resistance to each antibiotic was compared [**Methods**], Fig. 6B, Supplemental Figs 14–15, Supplemental Table 11).

ARGs targeting quinolones, which are among the top 3 prescribed classes of antibiotics in both small animal veterinary and human medicine [109–111], were differentially carried (β for dogs relative to cats = 1.58, *q* = 0.020; β relative to humans = 3.41, *q* = 0.518) (Fig. 6A–B). These included *qnrB4* (quinolone resistance protein [112]), which were only identified in dogs, and the *acr* multidrug efflux family, which was found in all hosts (Supplemental Table 10). We also observed distinctive resistance patterns to antibiotics prescribed less frequently to companion animals, such as ansamycins and pleuromutilins. For instance, ansamycin resistance ARGs were detected in all hosts but were significantly higher in dogs relative to cats (β = 1.12, *q* = 0.054; β relative to humans = 2.91, *q* = 0.518) (Supplemental Fig. 15). The ansamycin rifampin is prescribed to dogs off-label, but its use in veterinary medicine is limited due to its risk for resistance [113–115]. Here, we identified the rifampin-resistant beta-subunit of RNA polymerase (*rpoB*) almost exclusively in dogs (β relative to cats = 4.81, *q* ≤ 0.001; β relative to humans = 3.35, *q* = 0.97), which was previously observed in multidrug-resistant *Staphylococcus pseudintermedius* isolates from dogs and resistant *Mycobacterium tuberculosis* and *E. coli* in humans [116–118]. In the current study, the *rpoB* gene was carried by the dog-specific *Bifidobacterium criceti* and the dog-enriched *B. pseudolongum* (Supplemental Fig. 16). Our results are consistent with previous observations that host-specific carriage of microbes, including *Bifidobacterium* in companion animals, determines risk of AMR [107].

## Discussion

This study provides the largest integrated data resource for the companion animal gut microbiome to date, incorporating 2639 total shotgun metagenomes, 730 previously published and 1909 new to this work. As a consequence, we have been able to profile the companion animal microbiome at unprecedented resolution, identifying 2320 total taxa, 561 not detected in the human gut and 19 new to this study. These results particularly inform the evolutionary biology of the gut microbial communities of companion animals and humans, who are closely related through physical proximity, shared environments, diets, and disease phenotypes. They also uniquely detail differences in microbial evolution across hosts, evidenced by host niche specificity of strain genetics and phylogeny both between and within microbial species. A subset of these differences were functionally annotatable, including metabolic and antibiotic resistance gene carriage. This is thus the first study to explore microbial community and phylogenetic differences across companion animal and human gut microbiomes at this scale, vastly improving our understanding of companion animal gut community membership, diversity, and relationship with the human microbiome.

Although zoonotic pathogen transmission is closely studied, it is easy to overlook the much more frequent interplay of non-pathogenic microbial transmission among hosts, especially humans and companion animals with whom we closely interact [91, 92, 119]. Not only is this critical for understanding the long-term health of humans and animals alike, companion animals have more significant interactions with humans compared to other captive mammals, providing a more accurate model for understanding how domestication and close human contact influence the overall evolution of microbial communities. Here, we identified community-wide, host-specific evolution of gut commensals at the level of SGBs, which is highly suggestive of the historical frequency of transmission events. We quantified lineage-specific divergence for SGBs, and our results were consistent with a previous study that identified the average co-speciation rates of genus-level clades [120]. In both studies, *Streptococcus* and *Bacteroides* were phylogenetically less host specific relative to other microbes. Our study differed from the former, however, in that we identified SGBs within *Clostridium* and *Gemmiger* to have varying lineage specificities as well. In contrast, in the previous study, these genera were identified to have a single high and low co-speciation score, respectively. This underscores the importance of our study’s SGB-level taxonomic resolution, which allowed us to identify subspecies-level host adaptation. Lastly, we showed that these strain-level, phylogenetic differences manifested as gene differences across hosts likely driven by host-specific selective pressures [9, 120, 121], although future work incorporating non-domesticated, phylogenetically related host species would further delineate the evolution of the gut microbiome in cats and dogs [122].

We observed subspecies-level differences in the carriage of genes conferring resistance to several classes of antibiotics. Recent studies have investigated ARG carriage for several commensals at the genus, family, and order level or without stratifying across hosts [107, 123, 124]. In our study, we compared species-level diversity in ARG profiles specific to companion animal and human hosts and identified commensals with potential resistance to a range of antibiotic classes. The extent of host-specific selective pressures driving the transfer of ARGs through processes such as lateral gene transfer, and more broadly AMR diversity in commensals, is not yet known, but there is evidence for ARG differences even within companion animal species due to host behaviors (e.g. diet, environmental exposure, preferred dwelling location) [107, 125]. A limitation of our method is that direct sequence alignment can result in misassignment of antibiotic resistance function for ambiguous genes, as in any such study. Further investigation is needed to understand the rate at which such misidentifications occur and for which subsets of genes (e.g. those with single nucleotide polymorphisms or regulatory resistance variants). The current study adds to our understanding of host niche-specific ARG diversity in companion animals, which is important for future studies aiming to understand pet and pet owner transmission and, additionally, how gut communities in cats and dogs recover from antibiotic perturbations [126].

Although we found evidence for the overall historical frequency of commensal transmission between companion animals and humans via strain phylogeny, the current study lacks sampling from cohabitating pet owner–pet pairs, which is necessary to conclusively determine transmission. Additionally, the metagenomic resources from this work serve as a baseline providing essential reference data for future functional studies examining microbial activity. Identifying changes in microbial community transcriptional expression is important to understand host–microbe interactions, especially during disease states or in response to exposures such as medications or diet shifts. Future companion animal studies could therefore measure microbial function at scale by incorporating accompanying metatranscriptomics and metabolomics. More targeted studies could also evaluate host-specific selective pressures that drive the evolution of subclades to better understand the genetic underpinnings of host specificity described here. This could be accomplished through interventional studies that measure the response of the microbiome in companion animals after changes in diet or other perturbations to the community (e.g. antibiotics), or by similar experiments performed *in vitro*. Lastly, multi-study efforts that aim to determine how host or environmental variables shape the gut microbiome require consistent metadata across studies in order to calculate meta-analysis statistics, which are critical for successful translation of microbiome biomarkers [127]. Thus, thoroughly including population descriptors such as breed, detailed dietary information, medication use, cohabitation, and dwelling information (e.g. stray vs. housed) would help generalize conclusions regarding the companion animal gut microbiome.

The One Health approach is a crucial pillar of public and environmental health, addressing phenotypes emerging from the interactions between animals, humans, and the environment. This study contributes to this concept by providing the most expansive and detailed profile of the companion animal gut microbiome to date, greatly expanding the number of samples publicly available to the research community. This resource is necessary not only for extending knowledge from human microbiome science into companion animal health, but also for exploring the basic biology of how microbes evolve with their host over relatively short time scales and frequent exposures. As a One Health microbiome resource, both the dataset and initial findings can help guide future studies to better target diet, disease, and antibiotic use in the context of the companion animal gut, improving the lives of pets and their families.

## Supplementary Material

Supplemental_Figures_revised_ISME_secondrevision_wrae201

Supplemental_Tables_README_wrae201

## Data Availability

The raw sequencing data are available in the NCBI Short Read Archive database under BioProject accession numbers PRJNA1082665 and PRJNA925857, in addition to the existing projects listed in Supplementary Table 1. The data and scripts used to generate the figures in this manuscript are provided in the following repositories: https://huttenhower.sph.harvard.edu/petspooled2024 and https://biom-mass.org/ohmr.
