# Supplementary material for: Comprehensive profile of the companion animal gut microbiome integrating reference-based and reference-free methods
Source: ISME J. 2024 Oct 12;18(1):wrae201. doi: 10.1093/ismejo/wrae201 (PMC11523182; doi:10.1093/ismejo/wrae201)
Supplement: Supplemental_Figures_revised_ISME_secondrevision_wrae201 [file supplemental_figures_revised_isme_secondrevision_wrae201.docx]

**Supplement**

| **Study** | **PMID** | **Accession** | **Authors** | **Publication Year** | **Sequencer** | **Cats (number Individuals)** | **Dogs (number Individuals)** | **Number of samples** |
| --- | --- | --- | --- | --- | --- | --- | --- | --- |
| Dysbiosis in a canine model of human fistulizing Crohn's disease | 32730134 | PRJNA531201 | Maldonado-Contreras et al. | 2020 | NextSeq 500 (Illumina) | NA | 20 | 108 |
| Deep Illumina-based shotgun sequencing reveals dietary effects on the structure and function of the fecal microbiome of growing kittens | 25010839 | PRJEB4391 | Deusch et al. | 2014 | HiSeq 2000 (Illumina) | 12 | NA | 36 |
| A Longitudinal Study of the Feline Faecal Microbiome Identifies Changes into Early Adulthood Irrespective of Sexual Development | 26659594 | PRJEB9357 | Deusch et al. | 2015 | HiSeq 2000 (Illumina) | 30 | NA | 88 |
| Pre- and post-weaning diet alters the faecal metagenome in the cat with differences in vitamin and carbohydrate metabolism gene abundances | 27876765 | https://www.mg-rast.org/mgmain.html?mgpage=project&project=mgp13384 | Young et al. | 2016 | HiSeq 2000 (Illumina) | 20 | NA | 20 |
| Similarity of the dog and human gut microbiomes in gene content and response to diet | 29669589 | PRJEB20308 | Coelho et al. | 2018 | HiSeq 2500 (Illumina) | NA | 64 | 129 |
| Metagenomic dissection of the canine gut microbiota: insights into taxonomic, metabolic and nutritional features | 30680877 | PRJNA504009 | Alessandri et al. | 2019 | NextSeq 500 (Illumina) | NA | 2 | 2 |
| Metagenomic analysis revealed beneficial effects of probiotics in improving the composition and function of the gut microbiota in dogs with diarrhoea | 31021333 | PRJNA524271;  PRJNA481475 | Xu et al. | 2019 | HiSeq X Ten Sequencing System (Illumina) | NA | 40 | 80 |
| Diet-induced remission in chronic enteropathy is associated with altered microbial community structure and synthesis of secondary bile acids | 31472697 | PRJNA515316 | Wang et al. | 2019 | NextSeq 500 (Illumina) | NA | 18 | 53 |
| Rapid Reconstitution of the Fecal Microbiome after Extended Diet-Induced Changes Indicates a Stable Gut Microbiome in Healthy Adult Dogs | 32303546 | PRJEB34360 | Allaway et al. | 2020 | HiSeq 4000 (Illumina) | NA | 8 | 48 |
| Feces Metagenomes and Metagenome-Assembled Genome Sequences from Two Separate Dogs (Canis lupus familiaris) with Multiple Diarrheal Episodes | 33239463 | PRJNA655841 | Ateba et al. | 2020 | NovaSeq (Illumina) | NA | 1 | 1 |
| Whole-Genome Shotgun Metagenomic Sequencing Reveals Distinct Gut Microbiome Signatures of Obese Cats | 35467389 | PRJNA758898 | Ma et al. | 2022 | NovaSeq 6000 (Illumina) | 16 | NA | 16 |
| Heterogeneity of gut microbial responses in healthy household dogs transitioning from an extruded to a mildly cooked diet | 34249503 | PRJNA714112 | Tanprasertsuk et al. | 2021 | HiSeq 2500 (Illumina) | NA | 28 | 56 |
| Differences in the gut microbiomes of dogs and wolves: roles of antibiotics and starch | 33676490 | PRJNA702770 | Liu et al. | 2021 | HiSeq 2500 (Illumina) | NA | 5 | 5 |
| Geographically diverse canid sampling provides novel insights into pre-industrial microbiomes | 35506233 | PRJNA738608 | Yarlagadda et al. | 2022 | NovaSeq 6000 (Illumina) | NA | 84 | 88 |
| Private samples (HPN)* |  |  |  |  | HiSeq 2500 and 4000 (Illumina) |  |  | 1,909  (cats=207,  (dogs=1,702) |
| **Total from companion animals** |  |  |  |  |  | **133**  **Individual cats** | **1,378**  **Individual dogs** | **2,639**  **companion animal gut metagenomes** |
| HMP1-II  (baseline samples, no repeated measures) |  |  |  |  |  |  |  | 238 |
| Madagascar  (no repeated measures) |  |  |  |  |  |  |  | 112 |
| **# human gut metagenomes** |  |  |  |  |  |  |  | **350** |
| **Total # gut metagenomes** |  |  |  |  |  |  |  | **2,989** |

##### **Supplemental Table 1: Public and private data sources of cat, dog, and human gut metagenomic samples.** Studies varied in the number of shotgun metagenomic samples contributed. *244 of these samples are published under BioProject PRJNA925857 and are noted as “Diet Intervention Study 4” throughout the manuscript.


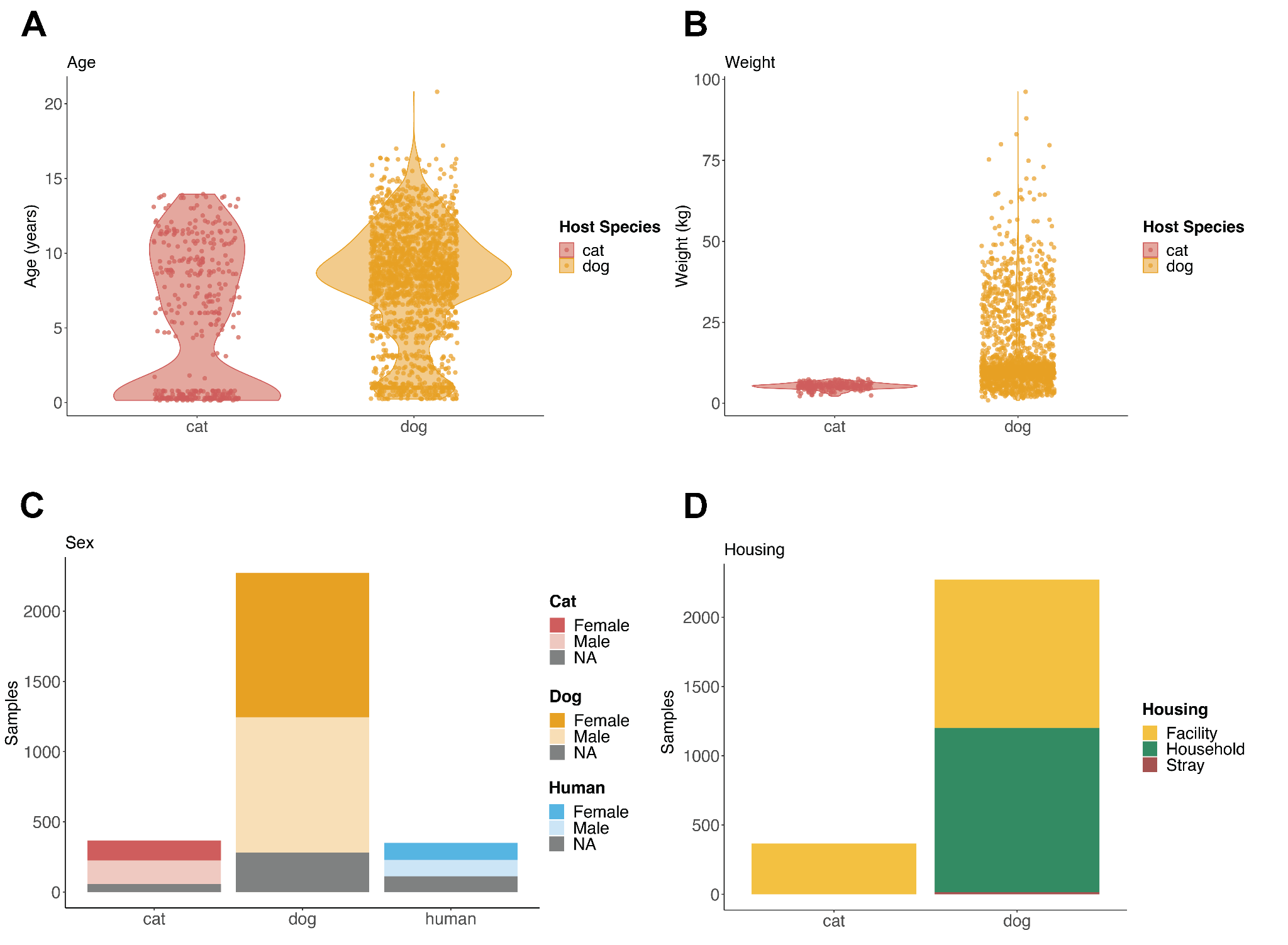


##### **Supplemental Figure 1: Companion animal metagenomic samples span various ages, weights, and housing conditions distribution.** Cats and dogs were 0.15 to 20.8 years old and weighed 0.9 to 96.2 kg. Most samples were accompanied with sex information (with an almost even split between males and females). Dogs were housed in either a facility or in private households, while the cats included in this study were only housed in facilities.


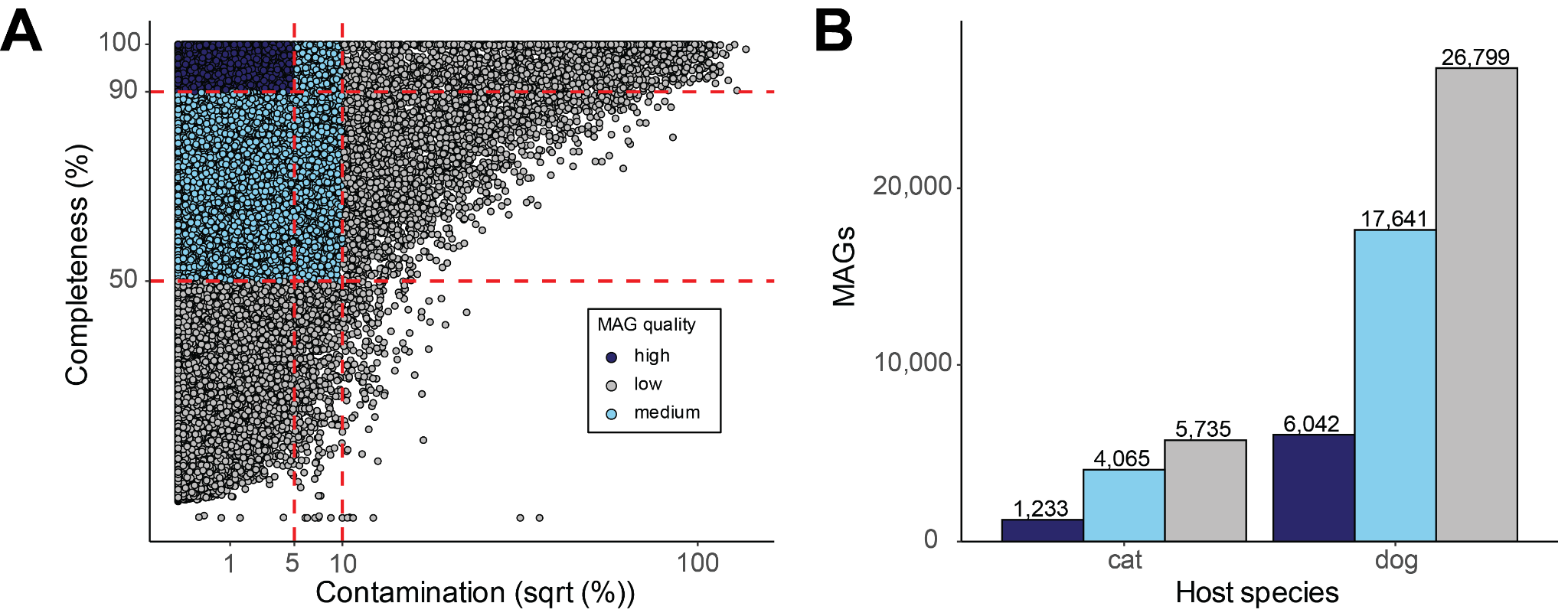


**Supplemental Figure 2: Quality determination of assembled MAGs from companion animals gut metagenomes.** **(A)** MAGs were categorized as high (>90% completeness and <5% contamination), medium (>50% completeness and <10% contamination), and low quality (<50% completeness and >10% contamination). **(B)** The high (1,233 cat and 6,042 dog) and medium quality (4,065 cat and 17,641 dog) MAGs from both cat and dog metagenomes were used for subsequent clustering into species-level genome bins (n = 28,981).


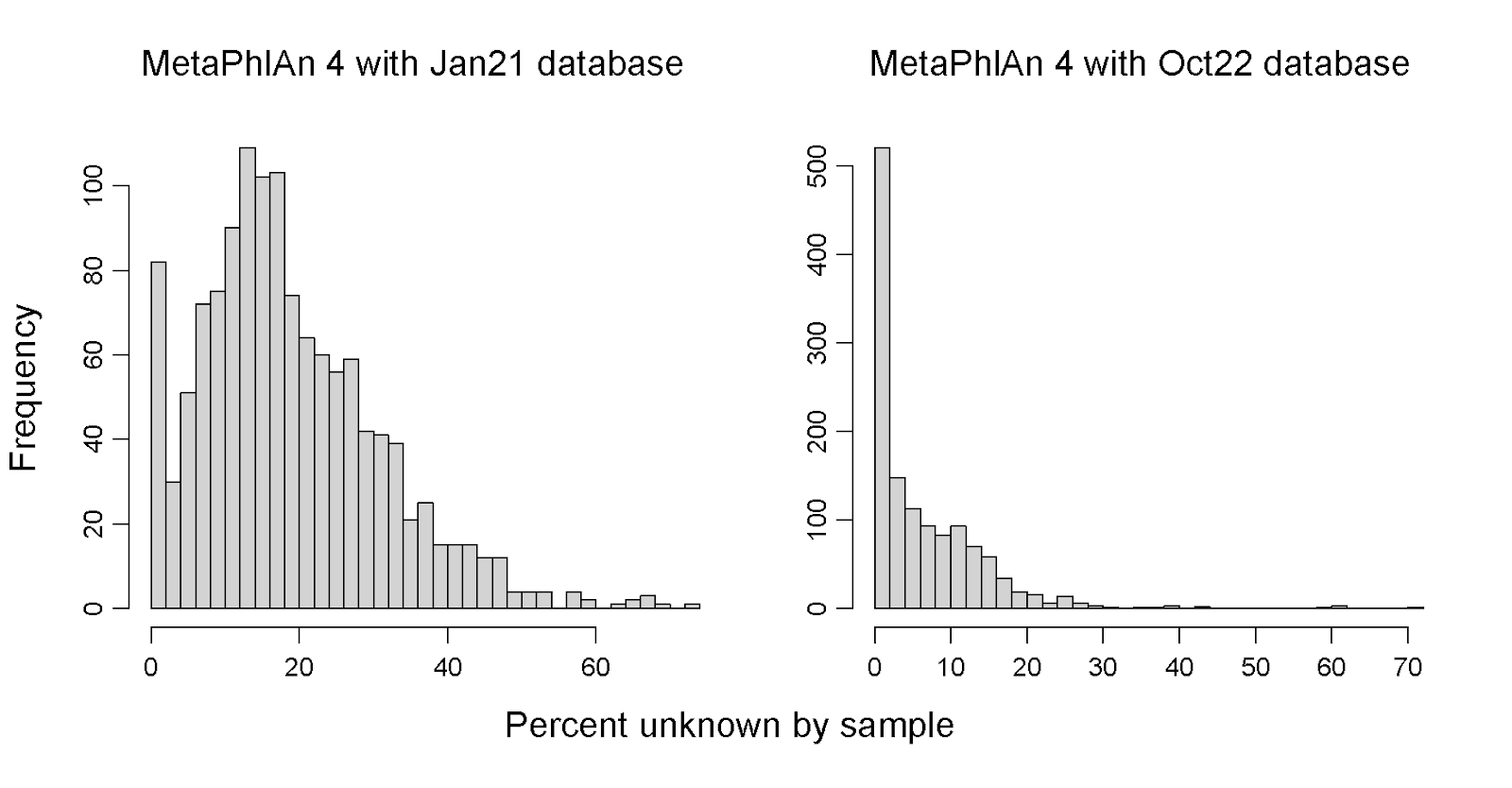
**Supplemental Figure 3: Incorporating the genomes assembled from companion animals gut metagenomes into a reference database improved taxonomic profiling with MetaPhlAn 4.** LEFT: The distribution of the percentage of reads, by sample, that did not contribute to taxonomic classification by MetaPhlAn 4 employing the Jan21 reference database (pre-update genomes assembled in our analysis from companion animal gut metagenomes). RIGHT: The distribution of the percentage of reads, by sample, that did not contribute to taxonomic classification by MetaPhlAn 4 employing the Oct22 reference database after the addition of the assembled genomes from our study’s companion animal gut metagenomes. Note, this plot does not include data from two publicly available studies (Coelho et al. and Yarlagadda et al.), as they were added to the analysis at a later time.


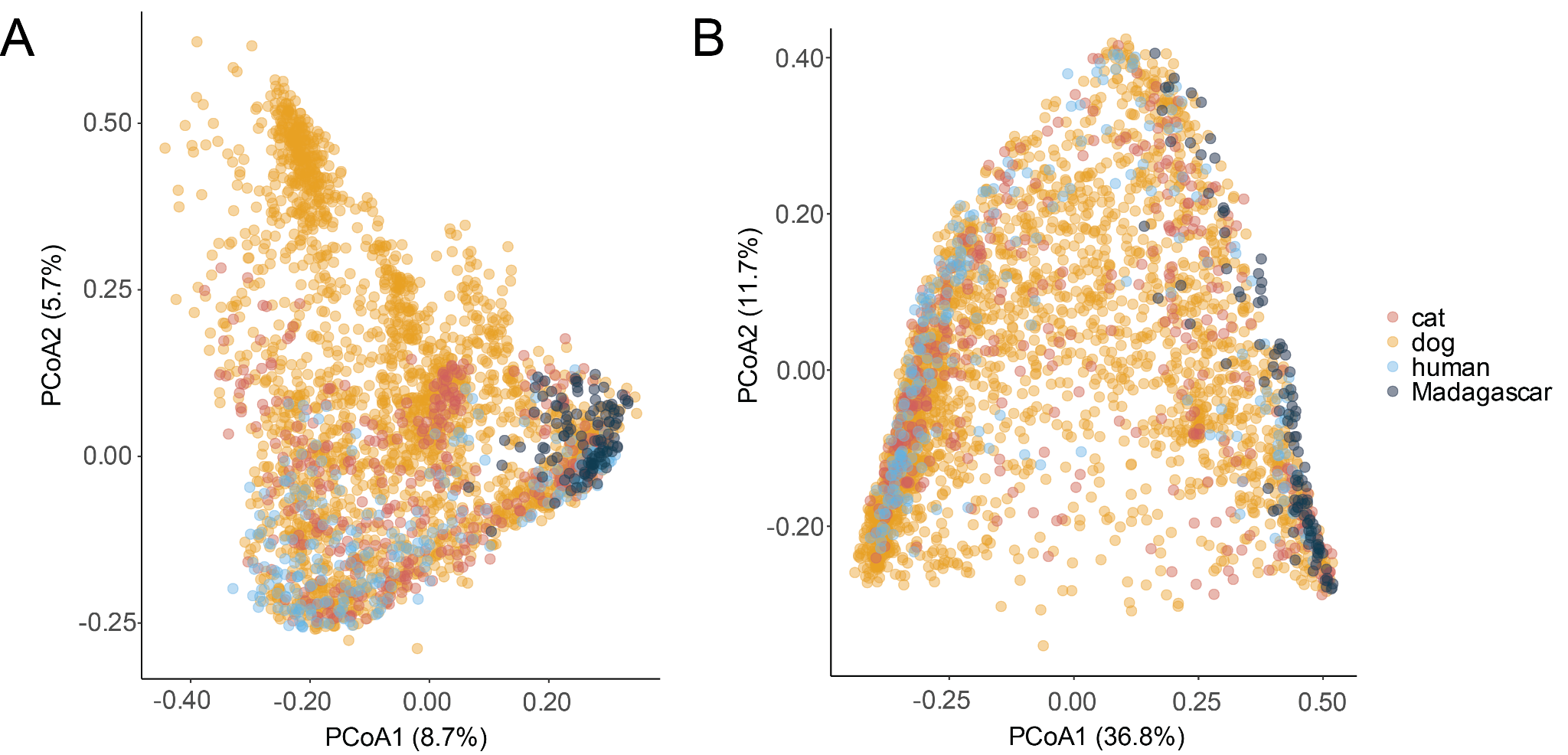


**Supplemental Figure 4. Phylogenetically aware methods demonstrate that overall phylogeny is similar across cat, dog, and human gut metagenomes.** Frequency-corrected principal coordinates analysis (PCoA) (by **(A)** unweighted and **(B)** weighted Unifrac dissimilarity) show the distribution of taxonomic profiles across hosts. PC scores were corrected by dividing the scores by the sample sizes of the respective host species.


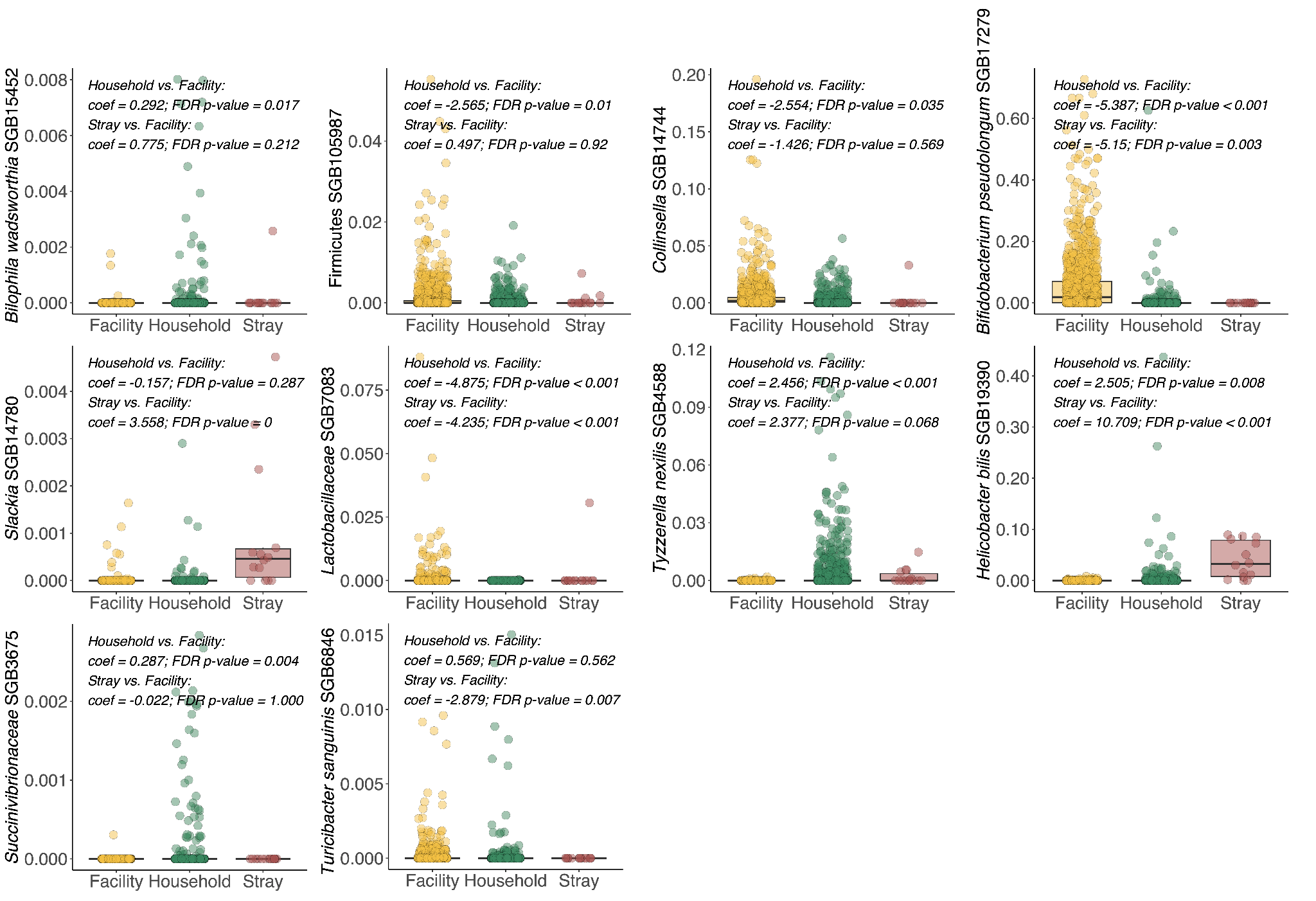


**Supplemental Figure 5: There are microbial differences in the composition of the gut microbiomes between dogs housed in facilities vs. private households.** Plots show the distribution of the relative abundance (log10 transformed) of microbes found in dogs in facilities and households. Effect sizes were calculated with MaAsLin 2, using “facility” as the reference value.


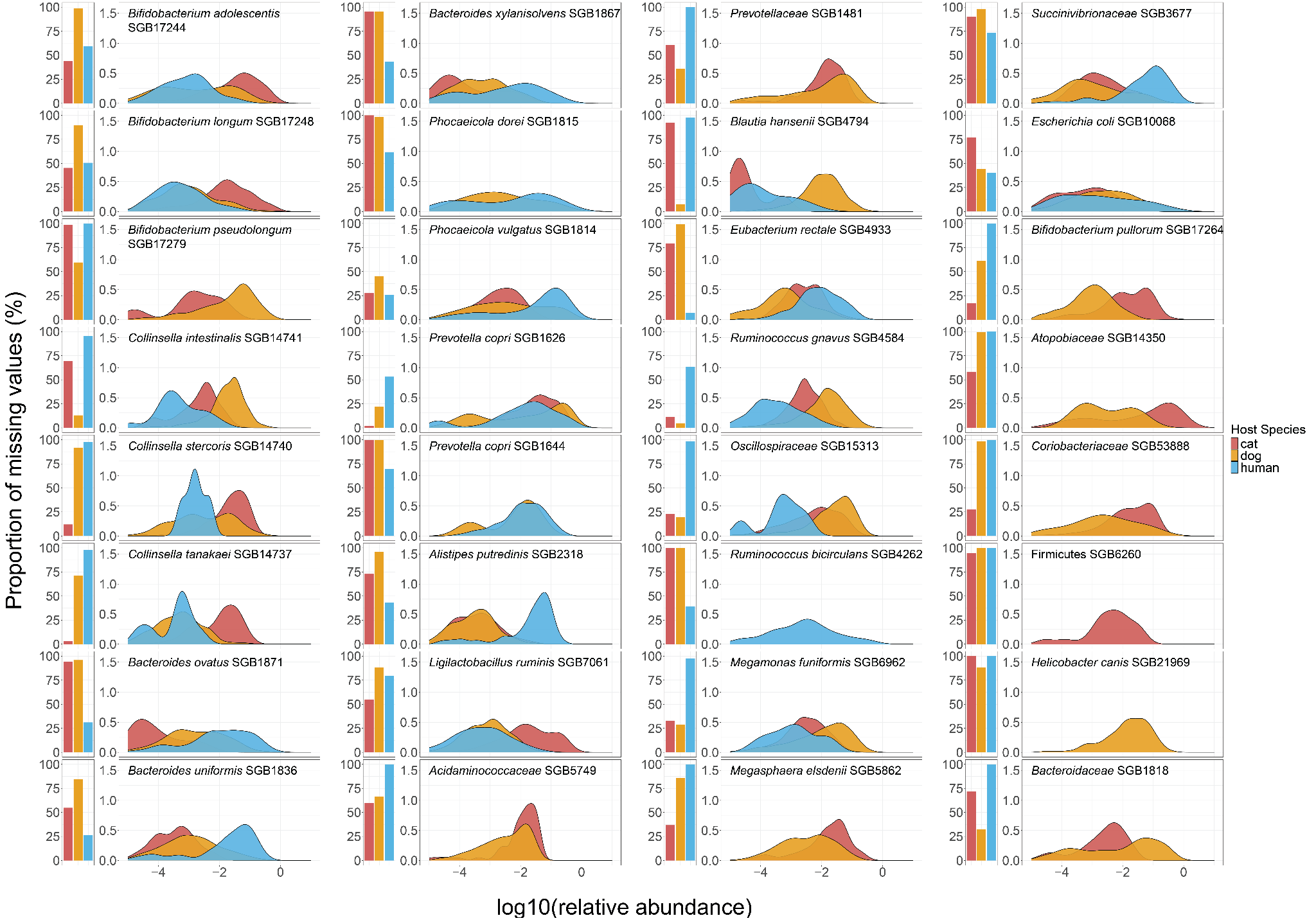


**Supplemental Figure 6: There are different distribution patterns of microbial species across host species.** Barplots show the proportion of samples that did not contain the respective SGB in each host. The right plots show the density distribution of relative abundance (log10 transformed). See abundance and prevalence filter criteria in **Methods**.


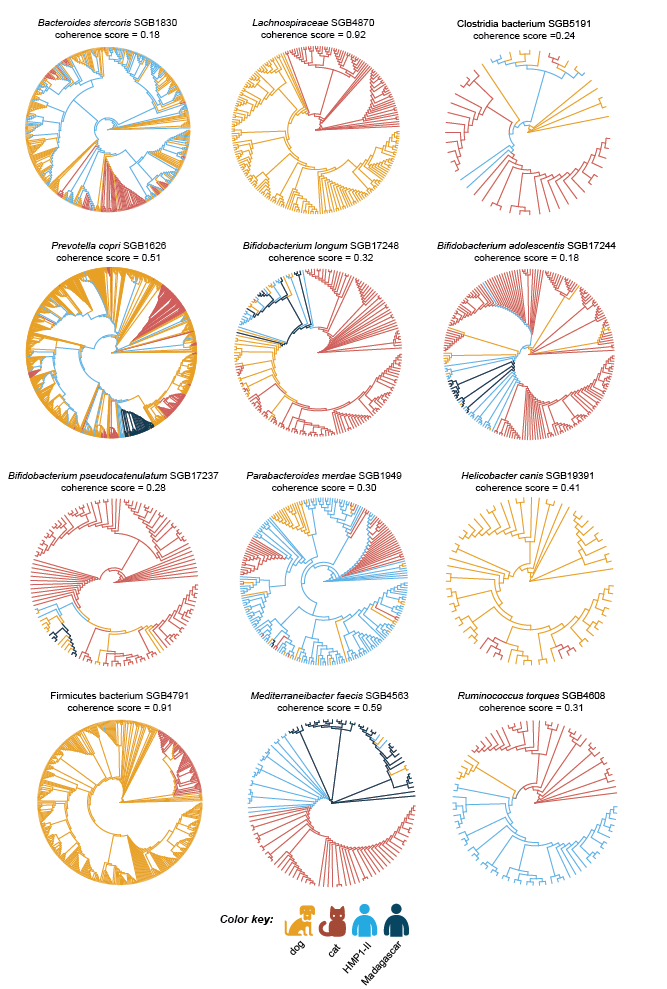


**Supplemental Figure 7**. **There are different phylogenetic distributions of sub-species clades for different SGBs**. For most microbes, strains distinctly cluster by host species (red = cat, yellow = dog, blue = human). Occasionally, single human or cat-recovered strains cluster with strains recovered from dogs, suggesting possible transmission events. In some cases, strains are universally similar across hosts, such as in *Bacteroides stercoris* SGB1830. Phylogenetic trees were built from the multiple sequence alignment derived using StrainPhlAn 4.


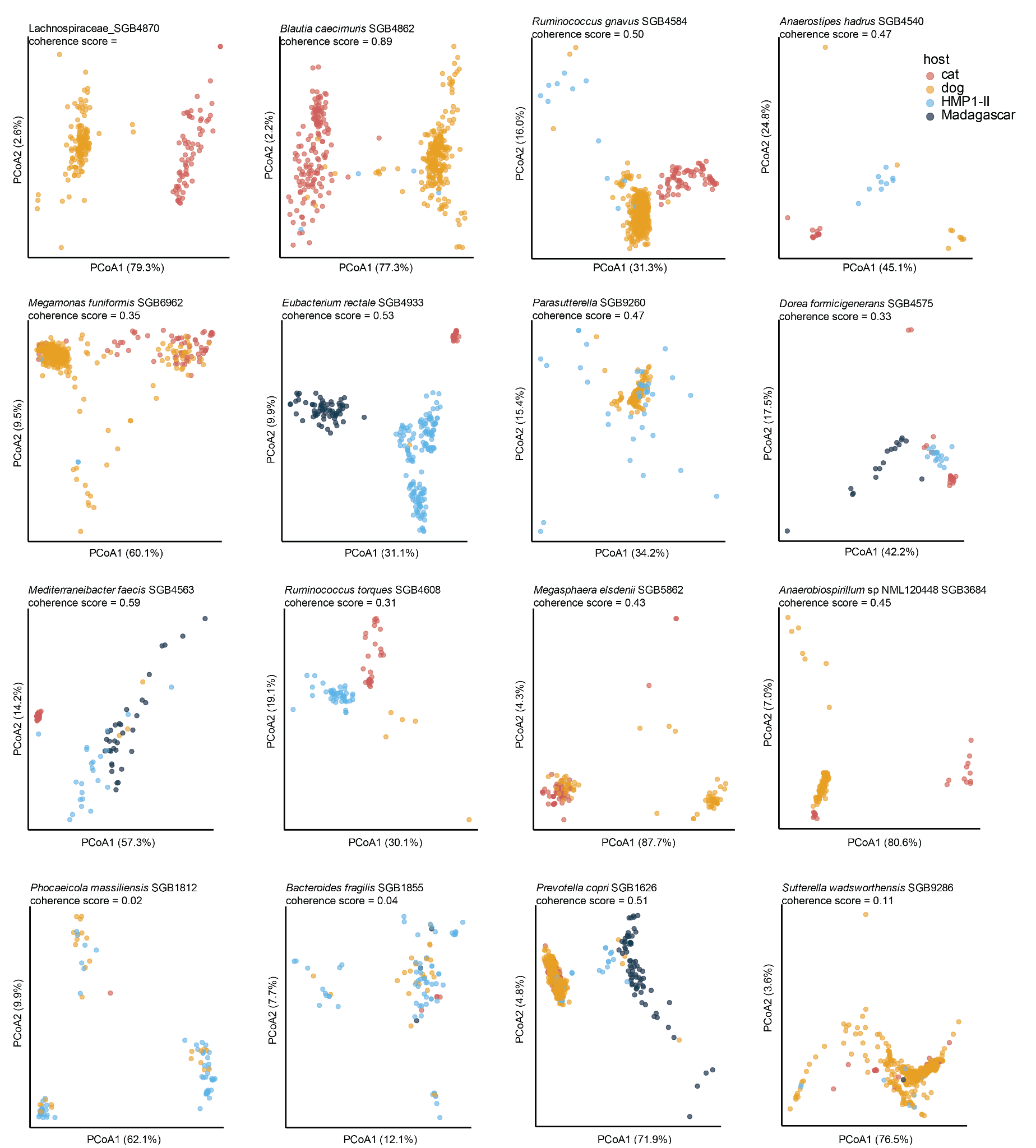


**Supplemental Figure 8: There is variability in the divergence of host-specific sub-clades across gut microbes.** Principal coordinate analyses of genetic distances (Kimura 2-parameter) calculated from multiple sequence alignments of subspecies generated using StrainPhlAn 4 (**Methods**).


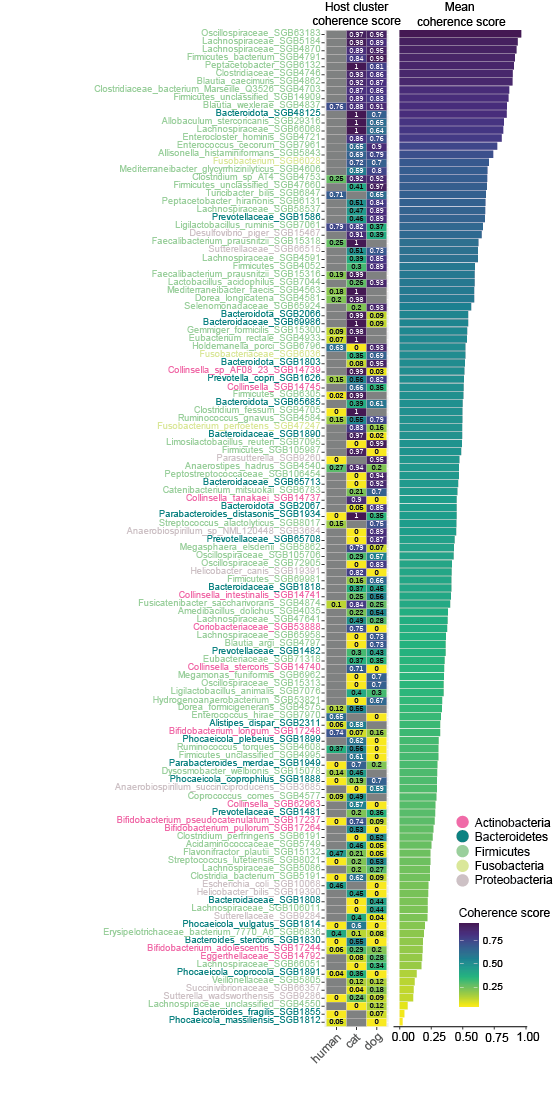


**Supplemental Figure 9: There is variability in lineage-specific divergence across SGBs.** Host-specific coherence scores and mean coherence score for each shared SGB (for shared SGBs with at least 5 samples passing the StrainPhlAn 4 coverage requirement, see **Methods**).


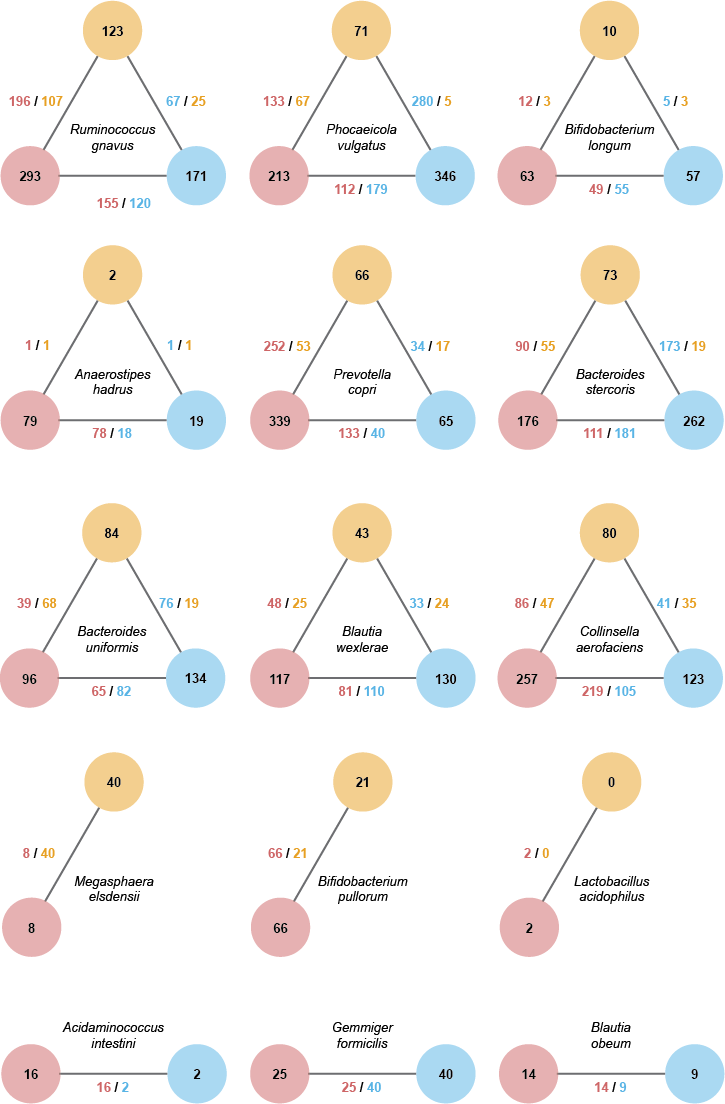


**Supplemental Figure 10. Strain-level gene carriage is different across host environments. (A)** Differential gene carriage in host-specific strains of shared microbes. For each microbial species, the inner edge values denote the total number of differentially carried genes by strains, as measured by anpan{https://github.com/biobakery/anpan}, for each pairwise comparison of hosts (effect sizes >= 1.0, qs <= 0.10) (**Methods**). Outer edge values refer to the number of significantly enriched genes in each host for that respective pairwise comparison (number of significantly enriched genes in pairwise tests are colored red for cats, yellow for dogs, and blue for humans). Node values signify the total number of significantly enriched genes carried by the respective microbe found in each host (the total number of genes unique to a host across pairwise host comparisons) (**Methods**). Values are presented as the number of genes per 1000 genes.


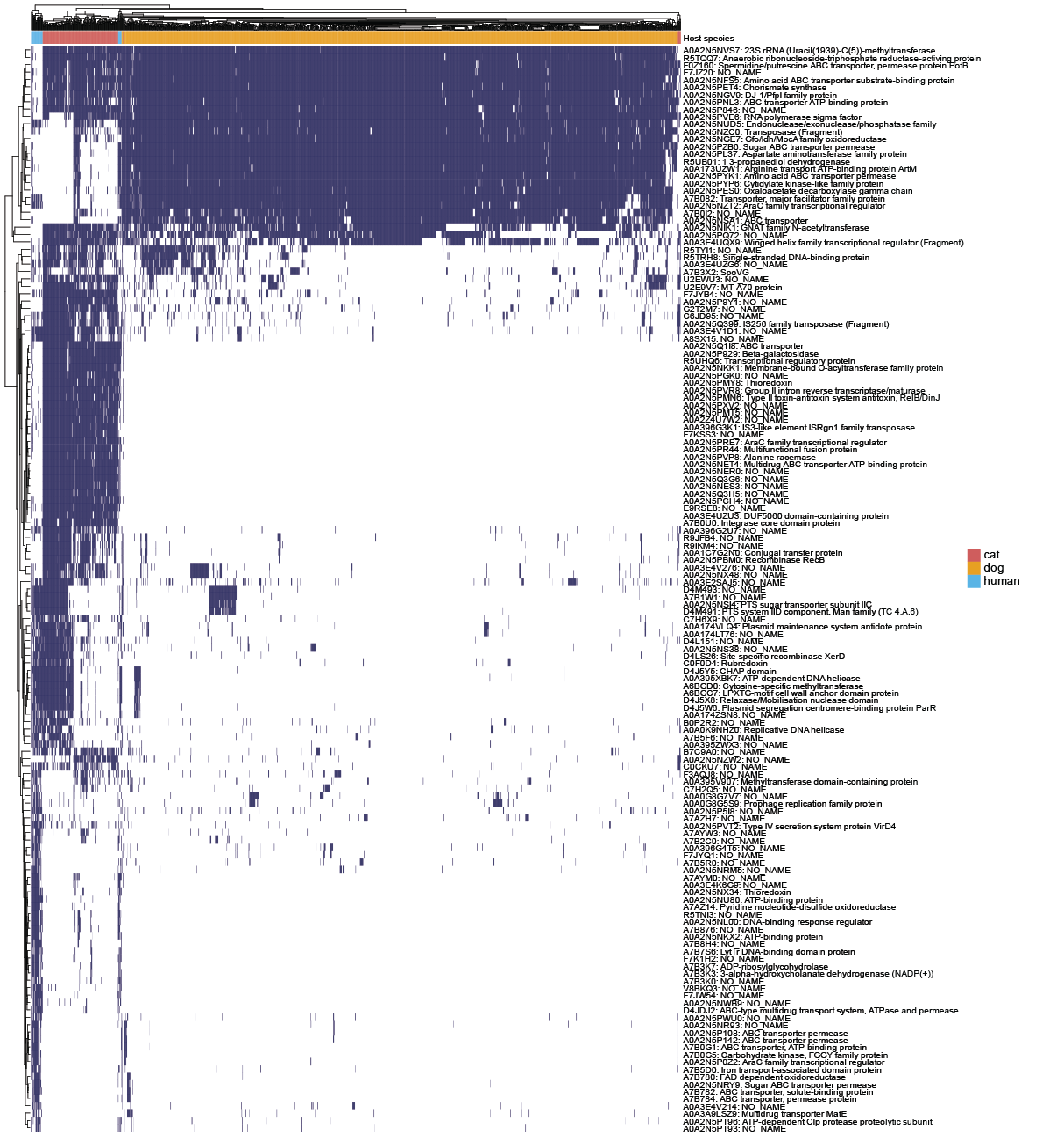


**Supplemental Figure 11**. **The functional capacity of *Ruminococcus gnavus* is host-dependent.** The heatmap shows the presence/absence of the most differential genes (rows, genes are annotated by the UniRef90 ID) measured for a pair of hosts, i.e. the 50 genes with the largest absolute value of the effect size for significantly different genes (q < 0.1) in each pairwise host comparison.


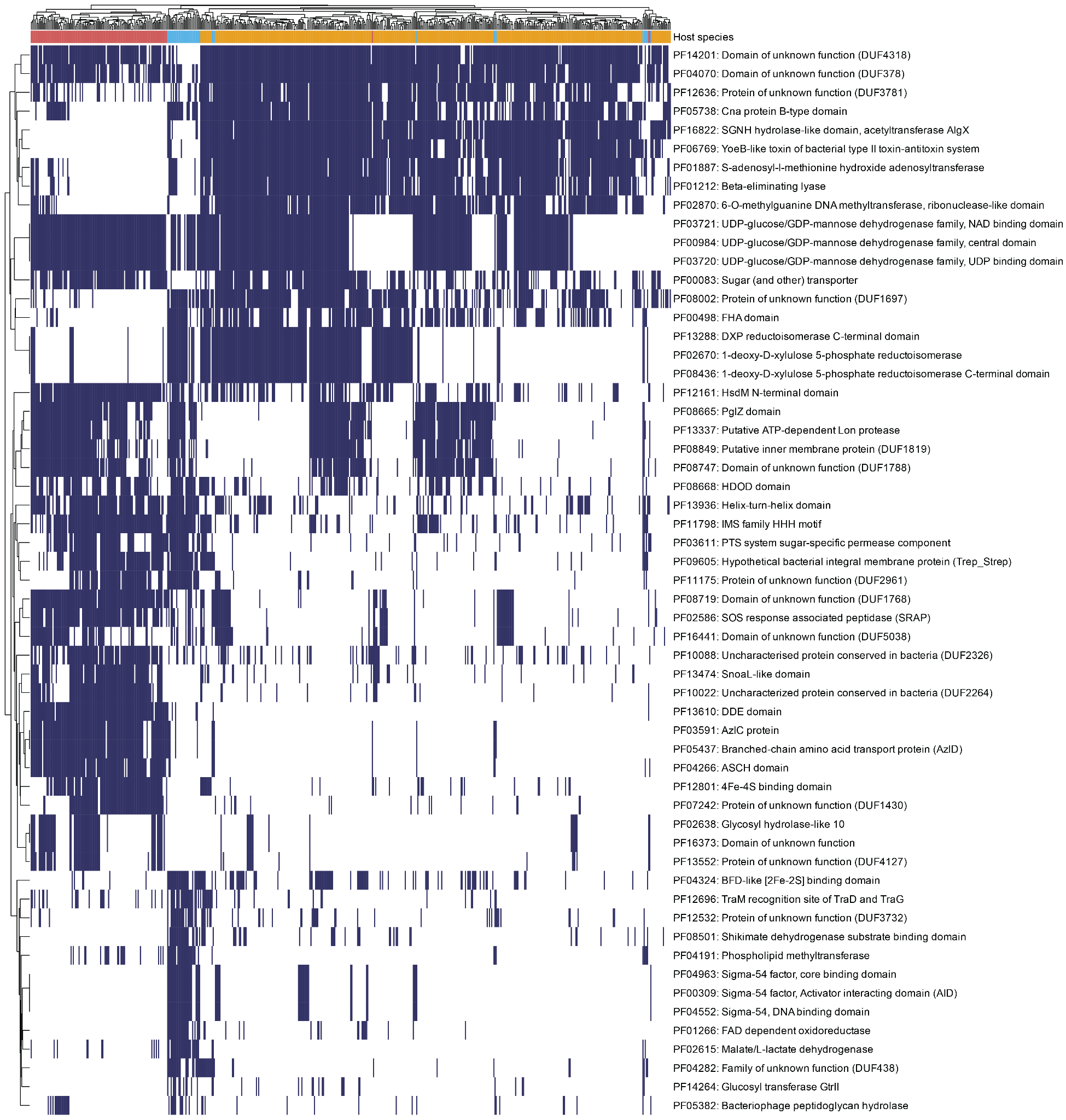


**Supplemental Figure 12**. **The functional capacity (protein families) of *Ruminococcus gnavus* is host-dependent.** The heatmap shows the presence/absence of the most differential protein families (rows, UniRef90 gene families were grouped into protein families) measured for a pair of hosts, i.e. the 20 genes with the largest absolute value of the effect size for significantly different genes (q < 0.1) in each pairwise host comparison.


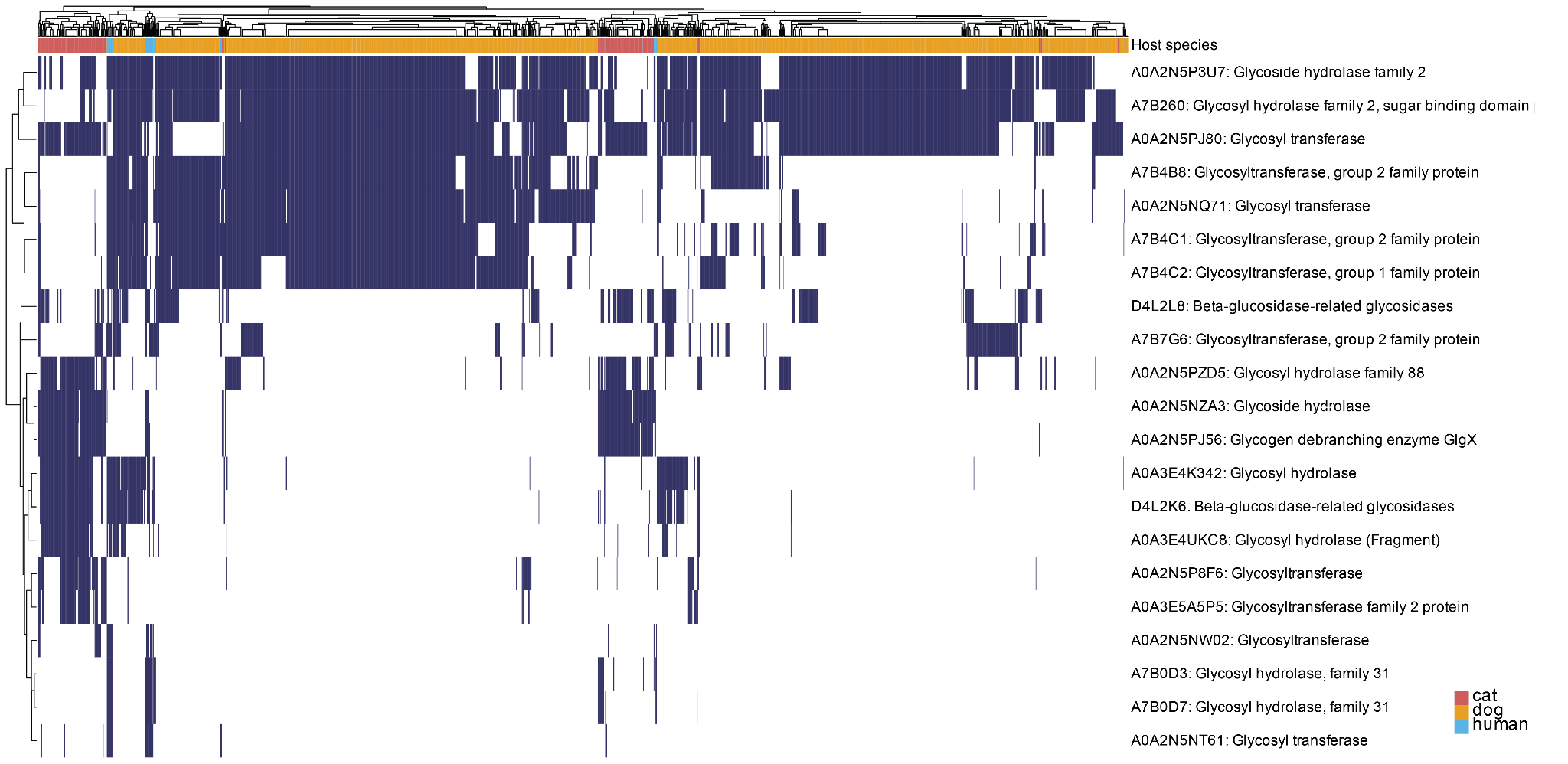


**Supplemental Figure 13**. ***Ruminococcus gnavus* carriage of glycosidase and glycotransferase-encoding genes is host-dependent.** The heatmap shows the presence/absence of the most differential glycosidase and glycotransferase-encoding genes (rows, genes are annotated by the UniRef90 ID) measured for a pair of hosts, i.e. among the 500 significantly different genes (q < 0.1) with the largest absolute effect sizes in each pairwise host comparison.


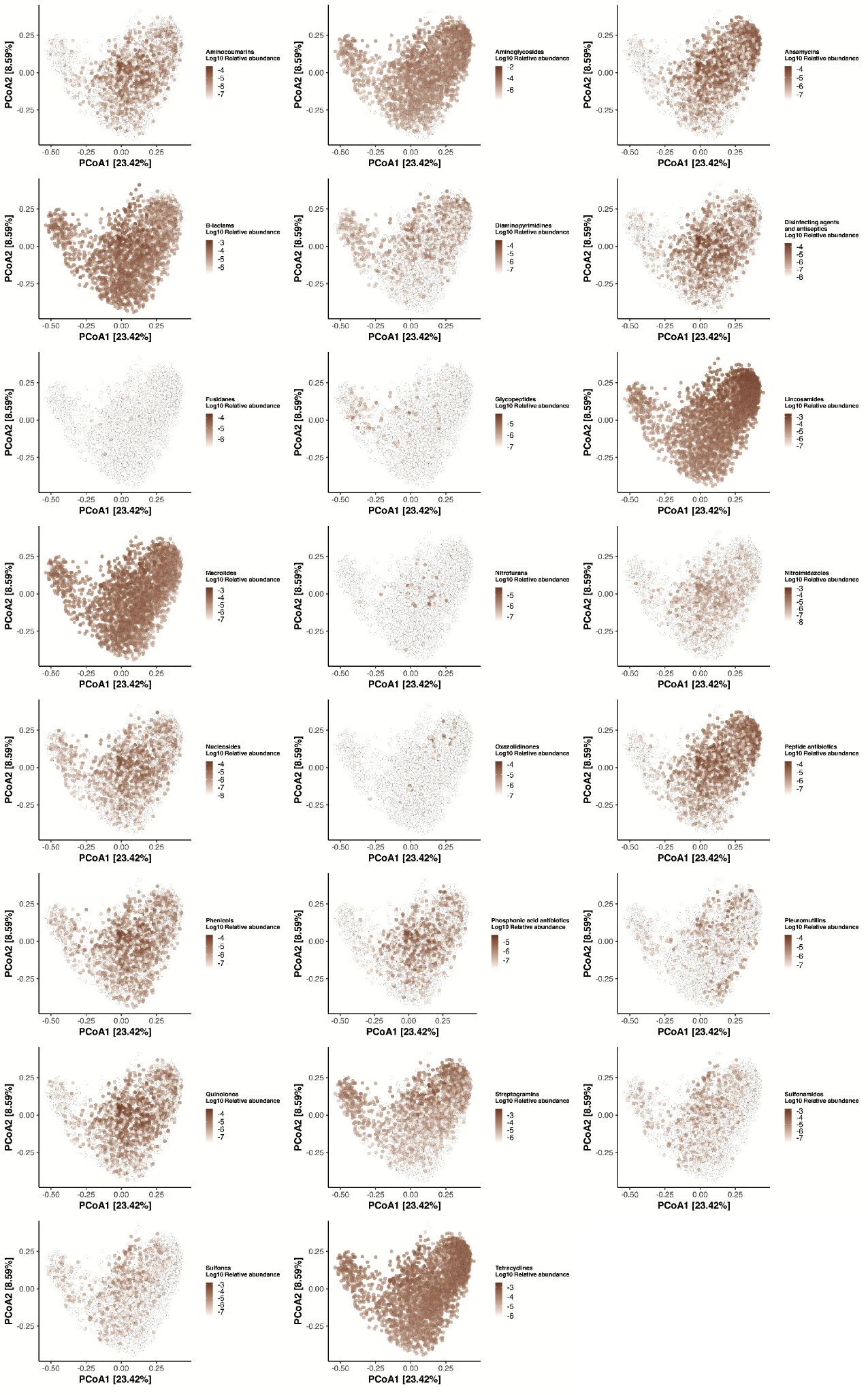


**Supplemental Figure 14. There is variability in the abundance of genes conferring resistance to diverse antibiotics across cat, dog, and human gut metagenomes.** Each plot is a PCoA of ARG profiles and the color overlay denotes the total abundance of ARGs that confer resistance to the respective antibiotic.


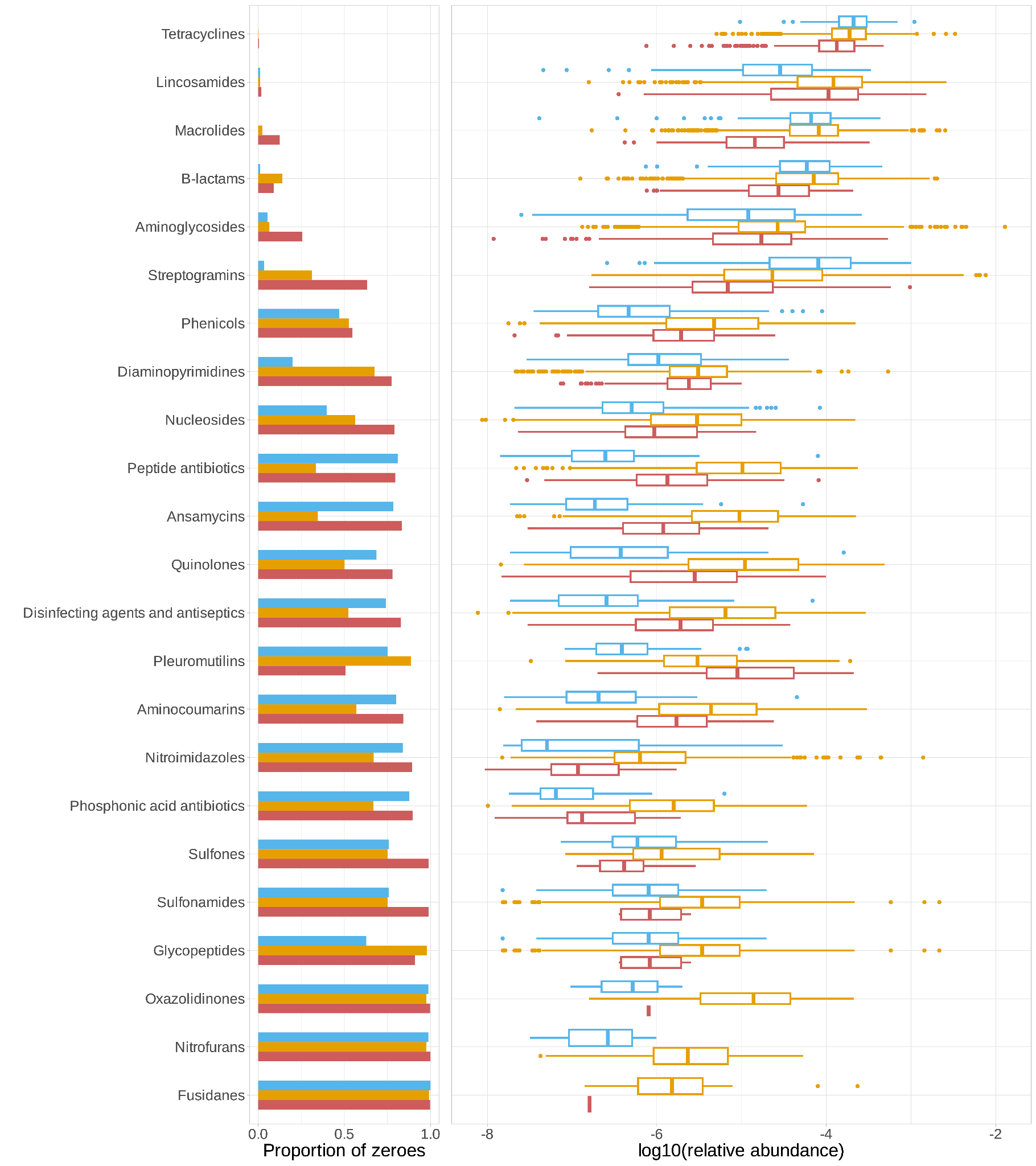


**Supplemental Figure 15. Gut microbiomes carry genes that confer resistance to diverse antibiotics.** Microbiomes carry a higher abundance of ARGs conferring resistance to commonly prescribed antibiotics (e.g., tetracyclines, B-lactams) relative to other antibiotics. LEFT: Proportion of samples, per host, without ARGs conferring resistance to the respective antibiotic (shown in rows). RIGHT: Total abundance of ARGs conferring resistance to the respective antibiotic. Microbiomes carry a higher abundance of ARGs conferring resistance to commonly prescribed antibiotics (e.g., tetracyclines, B-lactams) relative to other antibiotics.


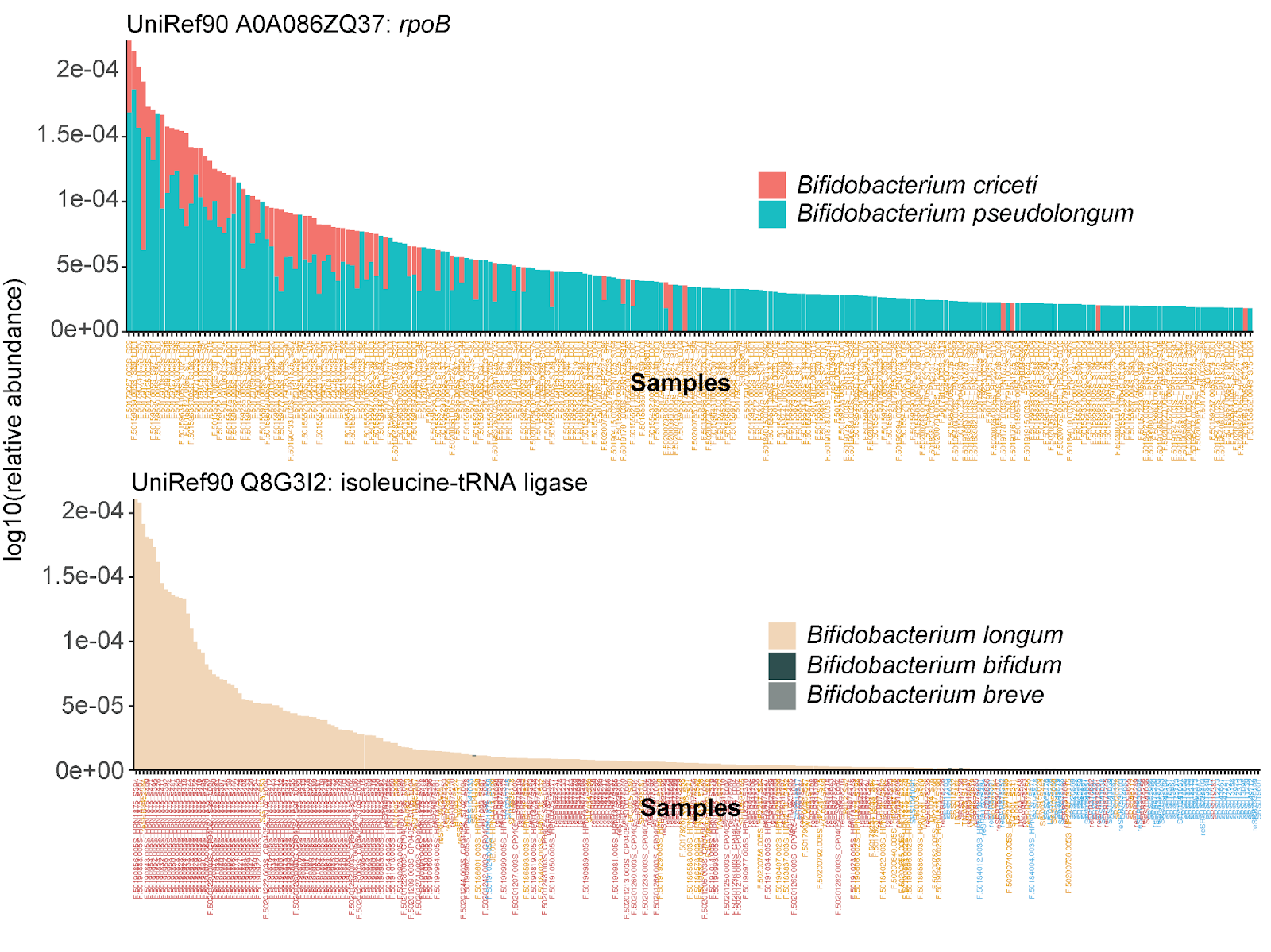


**Supplemental Figure 16. ARG carriage depends on carriage of microbes.** Barplots show the relative abundance (y-axis) of the rifampin-resistance conferring ARG *rpoB* (top) and the *ileS* ARG conferring resistance to mupirocin-like antibiotics (bottom) across samples (x-axis). Bars are colored by the microbial species that carried the ARG. Note, this plot does not include data from two publicly available studies (Coelho et al. and Yarlagadda et al.), as they were added to the analysis at a later time.
